# Risk for recurrent cardiovascular disease events among patients with diabetes and chronic kidney disease

**DOI:** 10.1186/s12933-021-01247-0

**Published:** 2021-03-01

**Authors:** Demetria Hubbard, Lisandro D. Colantonio, Robert S. Rosenson, Todd M. Brown, Elizabeth A. Jackson, Lei Huang, Kate K. Orroth, Stephanie Reading, Mark Woodward, Vera Bittner, Orlando M. Gutierrez, Monika M. Safford, Michael E. Farkouh, Paul Muntner

**Affiliations:** 1 Department of Epidemiology, University of Alabama At Birmingham, 1665 University Blvd, RPHB 140J, Birmingham, AL 35233-0013 USA; 2 Mount Sinai Heart, Icahn School of Medicine At Mount Sinai, New York, NY USA; 3 Department of Medicine, Division of Cardiovascular Disease, University of Alabama At Birmingham, Birmingham, AL USA; 4 Center for Observational Research, Amgen Inc., Thousand Oaks, CA USA; 5 The George Institute for Global Health, Imperial College, London, UK; 6 Department of Epidemiology and Biostatistics, School of Public Health, The George Institute for Global Health, University of New South Wales, Kensington, Australia; 7 Department of Epidemiology, Johns Hopkins University, Baltimore, MD USA; 8 Weill Cornell Medical College, Cornell University, Ithaca, NY USA; 9 Peter Munk Cardiac Centre, University of Toronto and Heart and Stroke Richard Lewar Centre of Excellence, Toronto, ON Canada

**Keywords:** Cardiovascular disease, Chronic kidney disease, Diabetes, Myocardial infarction, Risk factors, Epidemiology

## Abstract

**Background:**

Adults who have experienced multiple cardiovascular disease (CVD) events have a very high risk for additional events. Diabetes and chronic kidney disease (CKD) are each associated with an increased risk for recurrent CVD events following a myocardial infarction (MI).

**Methods:**

We compared the risk for recurrent CVD events among US adults with health insurance who were hospitalized for an MI between 2014 and 2017 and had (1) CVD prior to their MI but were free from diabetes or CKD (prior CVD), and those without CVD prior to their MI who had (2) diabetes only, (3) CKD only and (4) both diabetes and CKD. We followed patients from hospital discharge through December 31, 2018 for recurrent CVD events including coronary, stroke, and peripheral artery events.

**Results:**

Among 162,730 patients, 55.2% had prior CVD, and 28.3%, 8.3%, and 8.2% had diabetes only, CKD only, and both diabetes and CKD, respectively. The rate for recurrent CVD events per 1000 person-years was 135 among patients with prior CVD and 110, 124 and 171 among those with diabetes only, CKD only and both diabetes and CKD, respectively. Compared to patients with prior CVD, the multivariable-adjusted hazard ratio for recurrent CVD events was 0.92 (95%CI 0.90–0.95), 0.89 (95%CI: 0.85–0.93), and 1.18 (95%CI: 1.14–1.22) among those with diabetes only, CKD only, and both diabetes and CKD, respectively.

**Conclusion:**

Following MI, adults with both diabetes and CKD had a higher risk for recurrent CVD events compared to those with prior CVD without diabetes or CKD.

**Supplementary Information:**

The online version contains supplementary material available at 10.1186/s12933-021-01247-0.

## Background

Adults who have experienced multiple cardiovascular disease (CVD) events are at very high risk for recurrent events and are recommended intensive lipid-lowering therapy [1]. Diabetes mellitus and chronic kidney disease (CKD) are each associated with an increased risk for recurrent CVD events following a myocardial infarction (MI) [2–5]. Among adults without a prior history of CVD, the risk for incident CVD is higher for individuals with both diabetes and CKD versus their counterparts with neither, or only one, of these conditions [6]. There are few data on the risk for recurrent CVD events following an MI among patients who have both diabetes and CKD but have not had a prior CVD event. If patients with both diabetes and CKD have a similar or higher risk for recurrent CVD events after MI, compared to their counterparts with prior CVD, these individuals may benefit from intensive risk reduction interventions. 

The goal of this study was to compare the risk for recurrent CVD events and all-cause mortality, following hospital discharge for MI, among adults in four groups: (1) those with CVD prior to their MI without diabetes or CKD (referred to as prior CVD), and those without CVD prior to their MI who had (2) diabetes but not CKD (diabetes only), (3) CKD but not diabetes (CKD only) and (4) both diabetes and CKD. Also, previous studies have suggested that the risk for CVD events is higher for individuals with more versus less severe diabetes [7, 8]. Therefore, we repeated the analysis separating patients with diabetes into those taking and not taking insulin, a marker of diabetes severity [7, 8].

## Methods

We analyzed data from US adults with commercial health insurance through the MarketScan database and those with government health insurance through Medicare. We obtained MarketScan data for the calendar years 2006 through 2018 from Truven Health Analytics (IBM Watson Health). Medicare is a government program that provides health insurance for US adults ≥ 65 years of age and adults < 65 years of age with end-stage renal disease or who are disabled. We obtained data for all Medicare beneficiaries ≥ 65 years of age with fee-for-service, inpatient, outpatient, and pharmacy health insurance benefits who had an MI between 2006 and 2018 from the Centers for Medicare and Medicaid Services (CMS) Chronic Conditions Warehouse. The Institutional Review Board at the University of Alabama at Birmingham approved the study and waived the requirement to obtain informed consent.

### Study population

For the current analyses, we included patients who were hospitalized for an MI between January 1, 2014 and December 1, 2017. For each patient, we identified the discharge date for their first MI hospitalization on or after January 1, 2014 and used this as their index date for determining eligibility and the start of follow-up for outcomes. We restricted the study population to patients who had continuous fee-for-service inpatient, outpatient and pharmacy coverage and lived in the US for the 365 days prior to their index date (i.e., the “look-back” period) and were discharged alive. To avoid including the same MI hospitalization twice, we restricted the analyses to patients in the MarketScan database who were 19 to 64 years of age on their index date and patients in the Medicare database who were ≥ 66 years of age on their index date. We further restricted the sample to patients in the following four subgroups: (1) those with CVD prior to their index MI (prior CVD), and those without CVD prior to their index MI who had (2) diabetes only, (3) CKD only, and (4) diabetes and CKD (Additional file 1: Table S1). As the goal of this study was to compare event rates among those with prior CVD and their counterparts with diabetes and/or CKD without CVD, we excluded patients without prior CVD, diabetes or CKD and those who had prior CVD together with diabetes and/or CKD.

### Patient characteristics

We used all available claims between January 1, 2006 and each patient’s index date to identify prior CVD, diabetes, and CKD. Prior CVD included coronary heart disease (CHD), stroke and/or peripheral artery disease (PAD) events. The claims-based definitions used to define these conditions and diabetes and CKD are provided in Additional file 1: Table S2. The median time period available before each patient’s index date used to identify these conditions for patients included in this analysis was 2.45 years (25th, 75th percentiles: 1.33, 2.38 years).

We used beneficiary enrollment data on the index date to determine each patient’s age, sex, and geographic region of residence. Additionally, for Medicare patients, race/ethnicity was determined from the beneficiary enrollment data and area-level income was defined by the median income level within the beneficiary’s zip code of residence based on the 2017 American Community Survey [9]. Data on race/ethnicity and area-level income are not available in the MarketScan database. We used all available claims prior to each patient’s index date to determine whether they had hypertension, a history of heart failure, depression, a prior MI hospitalization or a coronary artery bypass (CABG) surgery or percutaneous coronary intervention (PCI) outside of an MI*.* We also determined whether patients had a CABG/PCI during their index MI. Claims data were also used to define smoking status, receipt of care from a cardiologist or endocrinologist, and use of insulin, antihypertensive medication, ezetimibe and statin within 365 days prior to each patient’s index MI. Definitions of these characteristics are provided in Additional file 1: Table S3.

### Study outcomes

Patients were followed for the primary outcome of a CVD event, including a recurrent MI, CHD, stroke or PAD event, as defined in Additional file 1: Table S4. Components of the primary CVD outcome were assessed as secondary outcomes, separately. CHD events included hospitalizations for recurrent MI or inpatient or outpatient coronary revascularization. Stroke events included hospitalizations for ischemic or hemorrhagic stroke. PAD events included hospitalizations for acute limb ischemia, peripheral artery revascularization or thrombolysis, or non-traumatic lower extremity amputation above the ankle. For patients in the Medicare database, all-cause mortality was a secondary outcome and was identified using Social Security Administration-validated death dates from the Medicare beneficiary summary file. Mortality data are not available in the MarketScan database.

### Statistical analysis

Summary statistics were calculated for patients with (1) prior CVD, (2) diabetes only, (3) CKD only, and (4) both diabetes and CKD. For patients in each of these four groups, we calculated the rate of CVD, recurrent MI, CHD, stroke, and PAD events. Also, we calculated the cumulative incidence of CVD, recurrent MI, CHD, stroke, and PAD events using the Kaplan–Meier method. Using Cox proportional hazards regression, we estimated hazard ratios (HR) and 95% confidence intervals (CI) for CVD events, recurrent MI, CHD, stroke, and PAD events for those with diabetes only, CKD only, and both diabetes and CKD compared to the reference group of patients with prior CVD. The first model included adjustment for age, sex, geographic region of residence, race/ethnicity, and area-level income. The second model included additional adjustment for smoking, hypertension, depression, history of heart failure, cardiologist care, endocrinologist care, antihypertensive medication use, statin therapy and intensity, and ezetimibe use. Among patients with Medicare health insurance, we calculated the rates, cumulative incidence, and HRs for all-cause mortality, as described for the CVD outcome. For all time-to-event analyses, patients were followed from their index date to the first occurrence of each outcome event, loss of fee-for-service inpatient or outpatient coverage, death for Medicare patients, or December 31, 2018.

Given previous findings of sex differences in the risk for recurrent CVD events following an MI, we made an a priori decision to include analyses for men and women separately, testing for effect modification by sex by adding interaction terms to the Cox models [10, 11]. To assess differences by diabetes severity, we separated patients with diabetes into those taking and not taking insulin into different groups, and repeated the analyses described above. All analyses were conducted using SAS v. 9.4 (SAS Institute Inc., Cary, NC).

## Results

A total of 162,730 patients were discharged from the hospital following an MI between January 1, 2014 and December 1, 2017 and met the inclusion criteria for the current analysis. Of these patients, 89,920 (55.2%) had prior CVD, and 46,032 (28.3%), 13,459 (8.3%), and 13,319 (8.2%) had diabetes only, CKD only, and both diabetes and CKD, respectively (Table 1). Patients with prior CVD were more likely to be taking a high-intensity statin than those with diabetes only, CKD only, and both diabetes and CKD.

**Table 1:** Patient characteristics following myocardial infarction stratified by prior cardiovascular disease, diabetes, and chronic kidney disease

	**Prior CVD (n = 89,920)**^**a**^	**Diabetes only (n = 46,032)**	**CKD only (n = 13,459)**	**Diabetes and CKD** **(n = 13,319)**
Age, years, mean (SD)	77.1 (10.3)	72.0 (11.3)	81.1 (9.7)	77.4 (8.9)
Cohort, %				
MarketScan	8.2	18.9	3.5	4.1
Medicare	91.8	81.1	96.5	95.9
Males, %	53.3	50.1	44.6	45.4
Race/ethnicity, %^b^				
Black, Non-hispanic	5.5	9.0	7.7	11.7
White, Non-hispanic	90.4	82.8	87.7	80.1
Other	4.0	8.0	4.7	8.1
Geographic region, %				
West	14.4	15.5	16.2	16.3
Midwest	24.8	24.4	28.3	26.5
Northeast	20.6	18.0	18.0	17.1
South	40.2	42.1	37.5	40.0
Area-level income, %^b^				
< $35,000	7.9	9.7	7.6	10.4
$35,000–$49,999	33.3	34.5	32.0	35.0
$50,000–$74,999	36.8	36.6	39.6	36.5
≥ $75,000	22.0	19.2	20.8	18.2
Smoking, %	49.3	40.2	41.2	40.1
Hypertension, %	92.8	90.6	93.5	97.8
History of heart failure, %	40.1	31.9	46.7	54.4
Depression, %	30.2	25.8	28.3	30.3
Prior MI hospitalization, %	18.9	0.0	0.0	0.0
Prior CABG/PCI outside of an MI event, %	5.2	0.0	0.0	0.0
CABG/PCI during the MI event related to the index date, %	52.0	66.5	45.7	51.3
Cardiologist care, %	57.7	29.6	24.5	23.5
Endocrinologist care, %	2.3	8.6	2.4	11.0
Use of insulin, %	0.0	25.0	0.0	40.0
Statin use and intensity, %				
No statin	39.6	51.3	63.1	41.0
Low	5.7	6.4	5.8	7.5
Moderate	34.9	32.4	25.5	38.4
High	19.8	9.9	5.6	13.1
Ezetimibe use, %	4.2	2.3	1.6	2.9
Antihypertensive medication use, %	93.0	91.4	92.5	95.7

*CABG* coronary artery bypass graft, *CKD* chronic kidney disease, *CVD* cardiovascular disease, *PCI* percutaneous coronary intervention, *MI* myocardial infarction, *SD* standard deviation

^a^This group included patients with prior cardiovascular disease without diabetes or chronic kidney disease**.** Patients with prior cardiovascular disease and diabetes or chronic kidney disease were excluded from the analysis

^b^Among Medicare beneficiaries only

### Risk for cardiovascular events

**Fig. 1:**
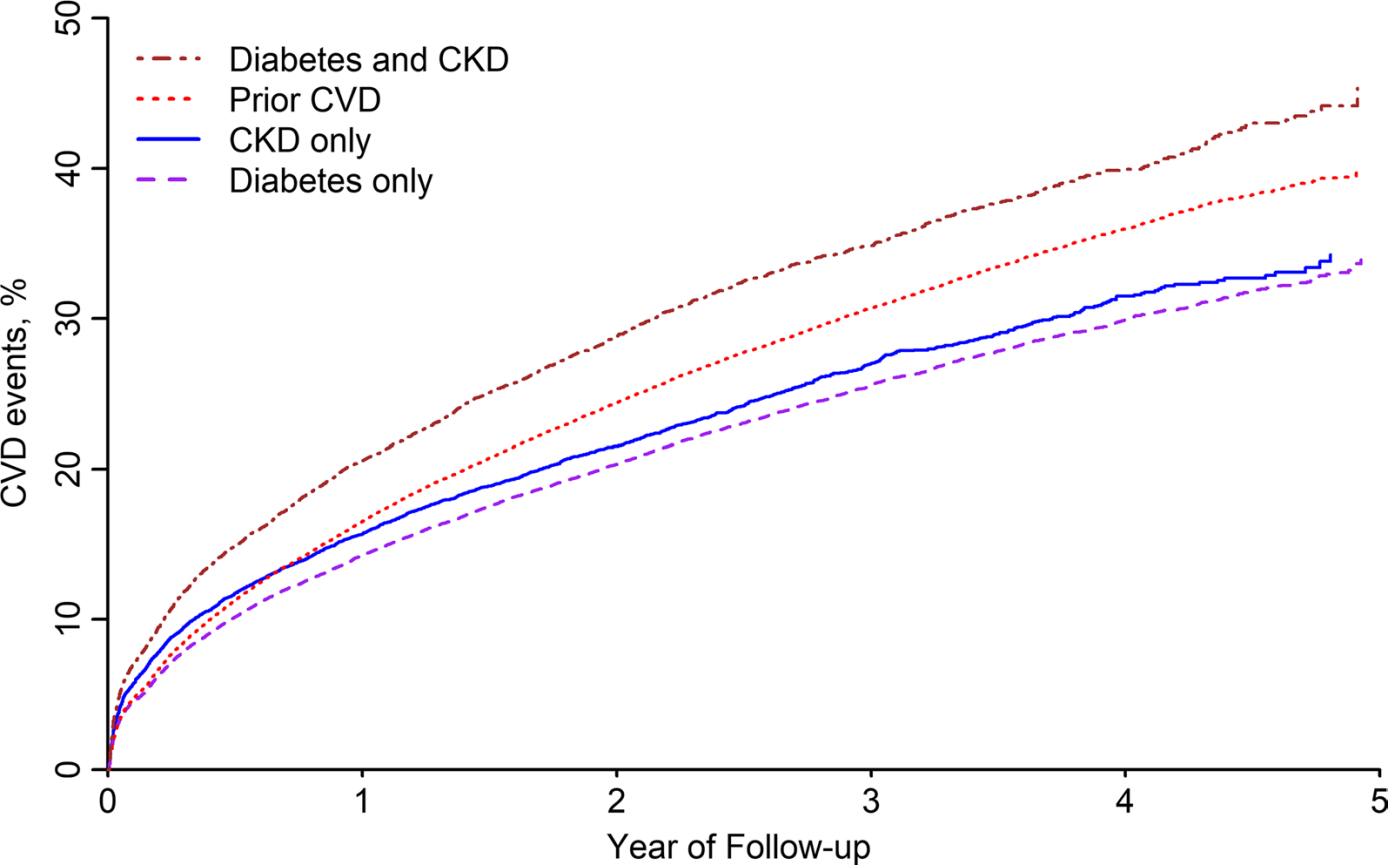
Cumulative incidence of cardiovascular disease events among patients following myocardial infarction.* CKD* chronic kidney disease,* CVD* cardiovascular disease

Compared to those with prior CVD, the cumulative incidence of CVD events was lower among patients with diabetes only and CKD only and higher among patients with both diabetes and CKD (Fig. 1). After full multivariable adjustment, compared to patients with prior CVD, the risk for CVD events was lower for patients with diabetes only (HR: 0.92; 95% CI: 0.90–0.95) and CKD only (HR: 0.89; 95% CI: 0.85–0.93) and higher for those with diabetes and CKD (HR: 1.18; 95% CI: 1.14–1.22) (Table 2). The risk for recurrent MI and CHD events was lower among patients with diabetes only and CKD only compared to those with prior CVD (Table 3 and Additional file 1: Figure S1). Patients with CKD only had a lower risk for stroke and PAD events compared to those with prior CVD. Patients with diabetes and CKD had a higher risk for recurrent MI, CHD, and PAD events when compared to those with prior CVD. There was no evidence of a difference in stroke risk between patients with diabetes and CKD compared to their counterparts with prior CVD.

**Table 2:** Risk for cardiovascular disease events following a myocardial infarction

	**Prior CVD (n = 89,920)**	**Diabetes only (n = 46,032)**	**CKD only (n = 13,459)**	**Diabetes and CKD (n = 13,319)**
Number of events	21,619	9390	2637	3517
Incidence rate (95% CI)	135 (133, 137)	110 (108, 112)	124 (119, 129)	171 (166, 177)
Hazard ratio (95% CI)				
Model 1	1 (ref)	0.86 (0.84–0.88)	0.86 (0.82–0.89)	1.20 (1.15–1.24)
Model 2	1 (ref)	0.92 (0.90–0.95)	0.89 (0.85–0.93)	1.18 (1.14–1.22)

*CI* confidence interval, *CKD* chronic kidney disease, *CVD* cardiovascular disease Incidence rates are presented as per 1000 person-years

Model 1 includes adjustment for age, sex, race/ethnicity (for patients in the Medicare sample), geographic region of residence and area-level income (for patients in the Medicare sample)

Model 2 includes adjustment for Model 1 plus smoking, hypertension, depression, history of heart failure, cardiologist care, endocrinologist care, antihypertensive medication use, statin therapy and intensity, and ezetimibe use

**Table 3:** Risk for myocardial infarction, coronary heart disease, stroke and peripheral artery disease following myocardial infarction

	**Prior CVD** **(n = 89,920)**	**Diabetes only** **(n = 46,032)**	**CKD only** **(n = 13,459)**	**Diabetes and CKD** **(n = 13,319)**
Myocardial infarction				
Number of events	14,350	5725	1894	2562
Incidence Rate (95% CI)	84 (82, 85)	63 (62, 64)	85 (81, 89)	117 (113, 122)
Hazard ratio (95% CI)				
Model 1	1.00 (ref)	0.83 (0.80–0.85)	0.89 (0.85–0.94)	1.30 (1.24–1.35)
Model 2	1.00 (ref)	0.88 (0.85–0.91)	0.92 (0.87–0.96)	1.25 (1.20–1.31)
Coronary heart disease				
Number of events	18,741	8055	2266	3051
Incidence Rate (95% CI)	114 (113, 116)	92 (90, 94)	105 (100, 109)	145 (140, 150)
Hazard ratio (95% CI)				
Model 1	1.00 (ref)	0.85 (0.82–0.87)	0.86 (0.82–0.90)	1.20 (1.16–1.25)
Model 2	1.00 (ref)	0.91 (0.88–0.94)	0.90 (0.86–0.94)	1.19 (1.14–1.24)
Stroke				
Number of events	3019	1342	401	469
Incidence Rate (95% CI)	16 (16, 17)	14 (13, 15)	17 (15, 18)	19 (18, 21)
Hazard ratio (95% CI)				
Model 1	1.00 (ref)	0.98 (0.91–1.04)	0.87 (0.79–0.97)	1.11 (1.01–1.23)
Model 2	1.00 (ref)	1.00 (0.93–1.08)	0.87 (0.78–0.97)	1.09 (0.98–1.20)
Peripheral artery disease				
Number of events	1238	577	104	241
Incidence Rate (95% CI)	7 (6, 7)	6 (5, 6)	4 (3, 5)	10 (9, 11)
Hazard ratio (95% CI)				
Model 1	1.00 (ref)	0.85 (0.77–0.94)	0.65 (0.53–0.79)	1.32 (1.15–1.52)
Model 2	1.00 (ref)	0.92 (0.83–1.03)	0.66 (0.54–0.81)	1.28 (1.11–1.49)

*CI* confidence interval, *CKD* chronic kidney disease, *CVD* cardiovascular disease Incidence rates are presented as per 1000 person-years

Model 1 includes adjustment for age, sex, race/ethnicity (for patients in the Medicare sample), geographic region of residence and area-level income (for patients in the Medicare sample)

Model 2 includes adjustment for Model 1 plus smoking, hypertension, depression, history of heart failure, cardiologist care, endocrinologist care, antihypertensive medication use, statin therapy and intensity, and ezetimibe use

### All-cause mortality

The risk for all-cause mortality was higher among patients with CKD only (HR: 1.03; 95% CI: 1.00–1.07) and diabetes and CKD (HR: 1.21; 95% CI: 1.18–1.25), and lower among those with diabetes only (HR: 0.89; 95% CI: 0.87–0.92), compared to those with prior CVD (Additional file 1: Table S5).

### Sex differences

There were statistically significant interactions by sex for CVD events, as well as recurrent MI, CHD, and all-cause mortality (p-values < 0.05, Additional file 1: Table S6). Among women, after multivariable adjustment, there was no evidence of a difference in the risk for CVD events between those with prior CVD and their counterparts with diabetes only and CKD only. Among men, diabetes only and CKD only were associated with a lower risk for CVD events when compared to prior CVD. The HR for CVD events associated with having diabetes and CKD versus prior CVD was 1.24 (95% CI: 1.17–1.30) among women and 1.12 (95% CI: 1.06–1.18) among men.

### History of diabetes and insulin use

Overall, 25% and 40% of patients with diabetes only and with both diabetes and CKD, respectively, were taking insulin. Compared to patients with prior CVD, those with diabetes only not taking insulin had a lower risk for CVD events (HR: 0.84; 95% CI 0.82, 0.87), while patients with diabetes only taking insulin had a higher risk for CVD events (HR: 1.20; 95% CI 1.15, 1.25) (Additional file 1: Table S7). The HR for CVD events was 1.06 (95% CI 1.02–1.11) and 1.42 (95% CI 1.35–1.49) among patients with diabetes and CKD not taking insulin and taking insulin, respectively, each versus those with prior CVD.

## Discussion

The 2018 American Heart Association/American College of Cardiology (AHA/ACC) multi-society cholesterol management guideline considers patients to have very high risk for recurrent CVD events if they have a history of multiple major CVD events or a history of one major CVD event and multiple high-risk conditions, including diabetes and CKD [1]. In the current study of patients discharged from the hospital after an MI, those with both diabetes and CKD and no prior CVD had a higher risk for recurrent CVD events compared to those with prior CVD without diabetes or CKD. They also had a higher risk for recurrent MI, CHD, and PAD events. Patients with diabetes only and CKD only had lower risk for recurrent CVD events when compared to their counterparts with prior CVD. However, when stratified by sex, women with diabetes only and CKD only had similar risk as compared to women with prior CVD.

Both diabetes and CKD have each been associated with an increased risk for recurrent CVD events [4, 12, 13]. Also, adults with both diabetes and CKD had a higher risk for cardiovascular events and mortality compared to their counterparts without diabetes or CKD, with diabetes without CKD, or with CKD without diabetes in the Jackson Heart Study [6]. In the REasons for Geographic and Racial Differences in Stroke (REGARDS) study, the risk for CHD events was lower among adults with diabetes without CHD versus their counterparts with CHD without diabetes [HR: 0.65; 95% CI: 0.54, 0.77] [8]. However, these Jackson Heart Study and REGARDS study analyses did not report the risk for recurrent CVD events among participants with diabetes only, CKD only and both diabetes and CKD versus their counterparts with a prior history of CVD. We excluded patients with a history of CVD who had diabetes and/or CKD, as estimating the risk for recurrent events in this population was beyond the scope of the current analysis. Patients with a history of CVD with diabetes and/or CKD are expected to have a higher risk for recurrent CVD events versus their counterparts with a history of CVD without diabetes or CKD according to prior studies [6, 7, 14–16].

CVD risk has been reported to differ by diabetes severity, which can be estimated using various measures including treatment intensity, diabetes duration, or comorbid CVD risk factors [8]. We used insulin therapy as an indicator of diabetes severity in the present analyses, as has been done previously [7, 8]. Among patients in the current study with diabetes only and diabetes and CKD, the risk for recurrent CVD events was higher for those taking versus not taking insulin. These findings suggest that diabetes severity should be evaluated when assessing the risk for recurrent CVD events among patients with diabetes.

Among men, diabetes only and CKD only were associated with a lower risk for recurrent CVD events versus prior CVD. However, there was no evidence of a difference in the risk for recurrent CVD events among women with diabetes only or CKD only versus with prior CVD. Also, the increased risk for recurrent CVD events among patients with diabetes and CKD versus those with prior CVD was larger among women versus men. In a previous meta-analysis, the increased risk for incident CVD associated with diabetes was larger among women compared with men (risk ratio 2.82; 95% CI: 2.35, 3.38, versus 2.16; 95% CI: 1.82, 2.56, respectively) [10]. Along with the results of the current study, these data suggest that the presence of CKD and diabetes may be associated with a greater excess CVD risk among women compared with men.

The 2018 AHA/ACC cholesterol guideline recommends that all adults with a history of CVD take a high-intensity, or maximally-tolerated, statin [1]. The guideline also recommends patients with very high risk for recurrent CVD events and low-density lipoprotein cholesterol (LDL-C) ≥ 70 mg/dL while taking a maximally-tolerated statin be considered for the addition of ezetimibe and a proprotein convertase subtilisin/kexin type 9 (PCSK9) inhibitor [1]. Prior studies have shown that a substantial proportion of patients with a history of CVD are not taking a high-intensity statin, and the proportion of patients taking a high-intensity statin is lower among those with diabetes or CKD versus their counterparts with a history of CVD, especially among women [17–20]. In the current study, a higher percentage of patients with prior CVD only were taking a high-intensity statin when compared to those with diabetes only, CKD only and diabetes and CKD. However, the proportion of patients taking a high-intensity statin was low in all groups. The current findings support the need to increase high-intensity statin use following hospital discharge for MI. In addition to high-intensity statins, ezetimibe and PCSK9 inhibitors, other medications, including sodium-glucose cotransporter 2 (SGLT-2) inhibitors, have been shown to have both cardiovascular and renoprotective benefits among high-risk patients with diabetes or CKD [21–26]. Also, glucagon-like peptide-1 receptor agonists have been shown to reduce the risk for CVD and CKD outcomes in patients with high CVD risk and diabetes [27–29]. Given the very high risk for recurrent CVD events among adults with both diabetes and CKD, more intensive secondary prevention treatment following an MI may result in a substantial absolute risk reduction [30, 31].

There are several strengths associated with the current study, including its large sample size and high degree of generalizability by inclusion of patients who had commercial and Medicare health insurance from across the US. The results of the current study should be interpreted in the context of potential and known limitations. We did not include patients without health insurance. Therefore, results may not be generalizable to patients without health insurance. We used claims-based algorithms to define a history of CVD, diabetes, and CKD, which may result in some misclassification. However, multiple studies have validated these claims-based algorithms [32–37]. Also, by relying on claims data, we were unable to differentiate between patients with type 1 and type 2 diabetes. We did not have data on cholesterol levels or information on statin intolerance.

## Conclusions

The results of the current study suggest that adults with both diabetes and CKD have a higher risk for recurrent CVD events after an MI compared to their counterparts with prior CVD without diabetes or CKD. Also, among patients with and without CKD, the risk for recurrent CVD events is higher among those with diabetes taking versus not taking insulin. These findings highlight the need for intensive risk reduction interventions following MI among patients with both diabetes and CKD.

## Supplementary Information

**Additional file 1: **Additional figures and tables.

## Data Availability

Data used in the current study are available from the CMS and Truven Health Analytics. Other study information is available from the corresponding author.

## Abbreviations

AHA/ACC: American Heart Association/American College of Cardiology
CABG: Coronary artery bypass graft
CHD: Coronary heart disease
CI: Confidence interval
CKD: Chronic kidney disease
CMS: Centers for Medicare and Medicaid Services
CVD: Cardiovascular disease
HR: Hazard ratio
MI: Myocardial infarction
PAD: Peripheral artery disease
PCI: Percutaneous coronary intervention
