# Supplementary material for: Risk for recurrent cardiovascular disease events among patients with diabetes and chronic kidney disease
Source: Cardiovasc Diabetol. 2021 Mar 1;20:58. doi: 10.1186/s12933-021-01247-0 (PMC7923492; doi:10.1186/s12933-021-01247-0)
Supplement: Supplementary file 1 — Additional file 1: Additional figures and tables. [file 12933_2021_1247_MOESM1_ESM.docx]

**Supplemental Materials: Risk for recurrent cardiovascular disease events among patients with diabetes and chronic kidney disease**

Demetria Hubbard,^1^ MSPH, Lisandro D. Colantonio,^1^ MD PhD, Robert S. Rosenson,^2^ MD, Todd M. Brown,^3^ MD MSPH, Elizabeth A. Jackson,^3^ MD MPH, Lei Huang,^1^ MS, Kate K. Orroth,^4^ PhD, Stephanie Reading,^4^ PhD, Mark Woodward, PhD,^5-7^ Vera Bittner,^3^ MD MSPH, Orlando M. Gutierrez,^1^ MD, Monika M Safford,^8^ MD, Michael E. Farkouh,^9^ MD MSc., Paul Muntner,^1^ PhD

^1^ Department of Epidemiology, University of Alabama at Birmingham, Birmingham AL, United States

^2^ Mount Sinai Heart, Icahn School of Medicine at Mount Sinai, New York NY, United States

^3^ Department of Medicine, Division of Cardiovascular Disease, University of Alabama at Birmingham, Birmingham AL, United States

^4^ Center for Observational Research, Amgen Inc., Thousand Oaks CA, United States

^5^ The George Institute for Global Health, University of Oxford, Oxford, United Kingdom

^6^ The George Institute for Global Health, University of New South Wales, Australia

^7^ Department of Epidemiology, Johns Hopkins University, Baltimore, MD

^8^ Weill Cornell Medical College, Cornell University, Ithaca, NY

^9^ Peter Munk Cardiac Centre, University of Toronto and Heart and Stroke Richard Lewar Centre of Excellence, Toronto ON, Canada

**Address for Correspondence:** Paul Muntner. 1665 University Blvd, RPHB 140J. Birmingham, AL 35233-0013. Phone: 205-975-8077. Fax: 205-975-7058. Email: [pmuntner@uab.edu](mailto:dhubbar8@uab.edu).

**Tables: 7**

**Figures: 1**

Supplemental Table 1. Inclusion and Exclusion Criteria by Conditions Present prior to the Index Myocardial Infarction

| **Conditions Present Prior to the Index Myocardial Infarction** | | |  |
| --- | --- | --- | --- |
| **CVD** | **Diabetes** | **CKD** | **Study Group** |
| Yes | No | No | Prior CVD |
| No | Yes | No | Diabetes Only |
| No | No | Yes | CKD Only |
| No | Yes | Yes | Both diabetes and CKD |
| Yes | Yes | No | Excluded |
| Yes | No | Yes | Excluded |
| Yes | Yes | Yes | Excluded |
| No | No | No | Excluded |

CKD: chronic kidney disease; CVD: cardiovascular disease.

Supplemental Table 2. Definitions for prior cardiovascular disease, chronic kidney disease, and diabetes.

|  | **Definition** |
| --- | --- |
| **Prior CVD** | Defined by having a history of coronary heart disease, stroke or peripheral artery disease, as described below. |
| History of coronary heart disease | Any of the following using all available claims prior to the admission for the MI hospitalization related to the index date:   - ≥1 hospitalization or physician evaluation and management visit with a diagnosis code of coronary heart disease (ICD-9-CM diagnosis code of 410.xx-414.xx or an ICD-10 code of ‘I200’, ‘I201’, ‘I208’, ‘I209’, ‘I21.xx’, ‘I22.xx’, ‘I240’, ‘I241’, ‘I248’, ‘I2510’, ‘I252’, ‘I253’, ‘I2541’, ‘I2542’, ‘I255’, ‘I25810’, ‘I25811’, ‘I25812’, ‘I2582’, ‘I2583’, ‘I2584’, ‘I2589’, ‘I259’, ‘Z95.1’ or ‘Z9861’) in any discharge diagnosis position - ≥1 hospitalization or physician visit with a procedure code for coronary revascularization (ICD-9-CM procedure codes 00.66, 36.0, 36.01-36.19, 36.2, ICD-10 procedure code starting with any of the following 4 digits: 0210, 0211, 0212, 0213, 0270, 0271, 0272, 0273, 02C0, 02C1, 02C2, 02C3, 3E07, or CPT codes 33510-33519, 33521-33523, 33530, 33533-33536, 92980-92982, 92984, 92995, 92996, 92920, 92921, 92924, 92925, 92928, 92929, 92933, 92934, 92937, 92938, 92941, 92943, 92944). |
| History of stroke | Any of the following using all available claims prior to the admission for the MI hospitalization related to the index date:   - ≥1 hospitalization with a discharge diagnosis code of stroke (ICD-9-CM discharge diagnosis code of 430.xx, 431.xx, 433.x1, 434.x1 or 436.x or ICD-10 codes of I60.xx, I61.xx, I63.xx and I67.89) in any discharge diagnosis position - ≥1 physician evaluation and management visit with a diagnosis code of stroke (ICD-9-CM discharge diagnosis code of 430.xx, 431.xx, 433.x1, 434.x1 or 436.x) in any position |
| History of peripheral artery disease | Any of the following using all available claims prior to the admission for the MI hospitalization related to the index date:   - ≥1 hospitalization with a discharge diagnosis code of atherosclerosis or thrombosis of arteries of the extremities (ICD-9-CM diagnosis code of 440.2, 440.20, 440.21, 440.22, 440.23, 440.24, 440.29, 440.3, 440.30, 440.31, 440.32, 440.4, 443.9 or ICD-10-CM diagnosis code of I70.2, I70.20, I70.201, I70.202, I70.203, I70.208, I70.209, I70.21, I70.211, I70.212, I70.213, I70.218, I70.219, I70.22, I70.221, I70.222, I70.223, I70.228, I70.229, I70.23, I70.231, I70.232, I70.233, I70.234, I70.235, I70.238, I70.239, I70.24, I70.241, I70.242, I70.243, I70.244, I70.245, I70.248, I70.249, I70.25, I70.26, I70.261, I70.262, I70.263, I70.268, I70.269, I70.29, I70.291, I70.292, I70.293, I70.298, I70.299, I70.3, I70.30, I70.301, I70.302, I70.303, I70.308, I70.309, I70.31, I70.311, I70.312, I70.313, I70.318, I70.319, I70.32, I70.321, I70.322, I70.323, I70.328, I70.329, I70.33, I70.331, I70.332, I70.333, I70.334, I70.335, I70.338, I70.339, I70.34, I70.341, I70.342, I70.343, I70.344, I70.345, I70.348, I70.349, I70.35, I70.36, I70.361, I70.362, I70.363, I70.368, I70.369, I70.39, I70.391, I70.392, I70.393, I70.398, I70.399, I70.4, I70.40, I70.401, I70.402, I70.403, I70.408, I70.409, I70.41, I70.411, I70.412, I70.413, I70.418, I70.419, I70.42, I70.421, I70.422, I70.423, I70.428, I70.429, I70.43, I70.431, I70.432, I70.433, I70.434, I70.435, I70.438, I70.439, I70.44, I70.441, I70.442, I70.443, I70.444, I70.445, I70.448, I70.449, I70.45, I70.46, I70.461, I70.462, I70.463, I70.468, I70.469, I70.49, I70.491, I70.492, I70.493, I70.498, I70.499, I70.5, I70.50, I70.501, I70.502, I70.503, I70.508, I70.509, I70.51, I70.511, I70.512, I70.513, I70.518, I70.519, I70.52, I70.521, I70.522, I70.523, I70.528, I70.529, I70.53, I70.531, I70.532, I70.533, I70.534, I70.535, I70.538, I70.539, I70.54, I70.541, I70.542, I70.543, I70.544, I70.545, I70.548, I70.549, I70.55, I70.56, I70.561, I70.562, I70.563, I70.568, I70.569, I70.59, I70.591, I70.592, I70.593, I70.598, I70.599, I70.6, I70.60, I70.601, I70.602, I70.603, I70.608, I70.609, I70.61, I70.611, I70.612, I70.613, I70.618, I70.619, I70.62, I70.621, I70.622, I70.623, I70.628, I70.629, I70.63, I70.631, I70.632, I70.633, I70.634, I70.635, I70.638, I70.639, I70.64, I70.641, I70.642, I70.643, I70.644, I70.645, I70.648, I70.649, I70.65, I70.66, I70.661, I70.662, I70.663, I70.668, I70.669, I70.69, I70.691, I70.692, I70.693, I70.698, I70.699, I70.7, I70.70, I70.701, I70.702, I70.703, I70.708, I70.709, I70.71, I70.711, I70.712, I70.713, I70.718, I70.719, I70.72, I70.721, I70.722, I70.723, I70.728, I70.729, I70.73, I70.731, I70.732, I70.733, I70.734, I70.735, I70.738, I70.739, I70.74, I70.741, I70.742, I70.743, I70.744, I70.745, I70.748, I70.749, I70.75, I70.76, I70.761, I70.762, I70.763, I70.768, I70.769, I70.79, I70.791, I70.792, I70.793, I70.798, I70.799, I70.9, I70.92 in any discharge diagnosis position - ≥2 physician evaluation and management visits with a diagnosis code of atherosclerosis or thrombosis of arteries of the extremities (ICD-9-CM diagnosis code of 440.2, 440.20, 440.21, 440.22, 440.23, 440.24, 440.29, 440.3, 440.30, 440.31, 440.32, 440.4, 443.9 or ICD-10-CM diagnosis code of I70.2, I70.20, I70.201, I70.202, I70.203, I70.208, I70.209, I70.21, I70.211, I70.212, I70.213, I70.218, I70.219, I70.22, I70.221, I70.222, I70.223, I70.228, I70.229, I70.23, I70.231, I70.232, I70.233, I70.234, I70.235, I70.238, I70.239, I70.24, I70.241, I70.242, I70.243, I70.244, I70.245, I70.248, I70.249, I70.25, I70.26, I70.261, I70.262, I70.263, I70.268, I70.269, I70.29, I70.291, I70.292, I70.293, I70.298, I70.299, I70.3, I70.30, I70.301, I70.302, I70.303, I70.308, I70.309, I70.31, I70.311, I70.312, I70.313, I70.318, I70.319, I70.32, I70.321, I70.322, I70.323, I70.328, I70.329, I70.33, I70.331, I70.332, I70.333, I70.334, I70.335, I70.338, I70.339, I70.34, I70.341, I70.342, I70.343, I70.344, I70.345, I70.348, I70.349, I70.35, I70.36, I70.361, I70.362, I70.363, I70.368, I70.369, I70.39, I70.391, I70.392, I70.393, I70.398, I70.399, I70.4, I70.40, I70.401, I70.402, I70.403, I70.408, I70.409, I70.41, I70.411, I70.412, I70.413, I70.418, I70.419, I70.42, I70.421, I70.422, I70.423, I70.428, I70.429, I70.43, I70.431, I70.432, I70.433, I70.434, I70.435, I70.438, I70.439, I70.44, I70.441, I70.442, I70.443, I70.444, I70.445, I70.448, I70.449, I70.45, I70.46, I70.461, I70.462, I70.463, I70.468, I70.469, I70.49, I70.491, I70.492, I70.493, I70.498, I70.499, I70.5, I70.50, I70.501, I70.502, I70.503, I70.508, I70.509, I70.51, I70.511, I70.512, I70.513, I70.518, I70.519, I70.52, I70.521, I70.522, I70.523, I70.528, I70.529, I70.53, I70.531, I70.532, I70.533, I70.534, I70.535, I70.538, I70.539, I70.54, I70.541, I70.542, I70.543, I70.544, I70.545, I70.548, I70.549, I70.55, I70.56, I70.561, I70.562, I70.563, I70.568, I70.569, I70.59, I70.591, I70.592, I70.593, I70.598, I70.599, I70.6, I70.60, I70.601, I70.602, I70.603, I70.608, I70.609, I70.61, I70.611, I70.612, I70.613, I70.618, I70.619, I70.62, I70.621, I70.622, I70.623, I70.628, I70.629, I70.63, I70.631, I70.632, I70.633, I70.634, I70.635, I70.638, I70.639, I70.64, I70.641, I70.642, I70.643, I70.644, I70.645, I70.648, I70.649, I70.65, I70.66, I70.661, I70.662, I70.663, I70.668, I70.669, I70.69, I70.691, I70.692, I70.693, I70.698, I70.699, I70.7, I70.70, I70.701, I70.702, I70.703, I70.708, I70.709, I70.71, I70.711, I70.712, I70.713, I70.718, I70.719, I70.72, I70.721, I70.722, I70.723, I70.728, I70.729, I70.73, I70.731, I70.732, I70.733, I70.734, I70.735, I70.738, I70.739, I70.74, I70.741, I70.742, I70.743, I70.744, I70.745, I70.748, I70.749, I70.75, I70.76, I70.761, I70.762, I70.763, I70.768, I70.769, I70.79, I70.791, I70.792, I70.793, I70.798, I70.799, I70.9, I70.92) in any discharge position on separate days - ≥1 hospitalization or physician visit with a CPT code 37205 or 75962 |
| **Chronic kidney disease** | Any of the following using all available claims prior to the index date, inclusive:   - ≥1 hospitalization with a discharge diagnosis code of chronic kidney disease (ICD-9-CM diagnosis code of 585.3 and 585.4 or ICD-10-CM diagnosis code of N18.3 and N18.4) in any discharge diagnosis position - ≥1 physician evaluation and management visit with a diagnosis code of chronic kidney disease (ICD-9-CM diagnosis code of 585.3 and 585.4 or ICD-10-CM diagnosis code of N18.3 and N18.4) in any position |
| **Diabetes** | Any of the following among patients without a history of diabetes mellitus using all available claims prior to the index date, inclusive:   - ≥1 hospitalization with a discharge diagnosis code of diabetes mellitus (ICD-9-CM diagnosis codes 250.xx, 357.2, 362.0x, or 366.41 or ICD-10-CM diagnosis codes 'E0836', 'E0842', 'E0936', 'E0942', 'E1010', 'E1011', 'E1029', 'E10311', 'E10319', 'E1036', 'E1039', 'E1040', 'E1042', 'E1051', 'E10618', 'E10620', 'E10621', 'E10622', 'E10628', 'E10630', 'E10638', 'E10641', 'E10649', 'E1065', 'E1069', 'E108', 'E109', 'E1100', 'E1101', 'E1129', 'E11311', 'E11319', 'E11329', 'E11339', 'E11349', 'E11359', 'E1136', 'E1139', 'E1140', 'E1142', 'E1151', 'E11618', 'E11620', 'E11621', 'E11622', 'E11628', 'E11630', 'E11638', 'E11641', 'E11649', 'E1165', 'E1169', 'E118', 'E119', 'E1310', 'E1336', 'E1342) in any discharge diagnosis position. - ≥2 physician evaluation and management visits with a diagnosis code of diabetes mellitus (ICD-9-CM diagnosis codes 250.xx, 357.2, 362.0x, or 366.41 or ICD-10-CM diagnosis codes 'E0836', 'E0842', 'E0936', 'E0942', 'E1010', 'E1011', 'E1029', 'E10311', 'E10319', 'E1036', 'E1039', 'E1040', 'E1042', 'E1051', 'E10618', 'E10620', 'E10621', 'E10622', 'E10628', 'E10630', 'E10638', 'E10641', 'E10649', 'E1065', 'E1069', 'E108', 'E109', 'E1100', 'E1101', 'E1129', 'E11311', 'E11319', 'E11329', 'E11339', 'E11349', 'E11359', 'E1136', 'E1139', 'E1140', 'E1142', 'E1151', 'E11618', 'E11620', 'E11621', 'E11622', 'E11628', 'E11630', 'E11638', 'E11641', 'E11649', 'E1165', 'E1169', 'E118', 'E119', 'E1310', 'E1336', 'E1342) in any position occurring at least 7 days apart. - ≥1 pharmacy claim for an oral hypoglycemic medicine, insulin, or non-insulin injectable hypoglycemic medicine; including glucagon-like peptide-1 (GLP-1) agonists, sodium-glucose co-transporter 2 (SGLT2) inhibitors, [repaglinide](https://www.uptodate.com/contents/repaglinide-drug-information?topicRef=1790&source=see_link), [pioglitazone](https://www.uptodate.com/contents/pioglitazone-drug-information?topicRef=1790&source=see_link), and dipeptidyl peptidase-4 (DPP-4) inhibitors |
| CPT: Current Procedure Terminology; HCPSC: Healthcare Common Procedure Coding System; ICD-9-CM: International Classification of Diseases, Ninth Revision, Clinical Modification; ICD-10-CM: International Classification of Diseases, Tenth Revision, Clinical Modification; MI: myocardial infarction.  In Medicare, physician evaluation and management visits were defined by a CPT code 99024, 99058, 99429, 99499, 99201-99215, 99241-99245, 99271-99275, 99301-99337, 99341-99355, 99385-99387, or 99395-99404, or 99281-99285. In MarketScan, physician evaluation and management visits were defined by an encounter type “AV”, “NH” or “ED”. | |

Supplemental Table 3. Definitions for patient characteristics and comorbid conditions.

|  | **Definitions** |
| --- | --- |
| Age | Identified using beneficiary enrollment data on their index date. |
| Sex | Identified using beneficiary enrollment data on their index date. |
| Race/ethnicity (among Medicare patients only) | Identified using beneficiary enrollment data on their index date. |
| Geographic region of residence | Defined based on the state of residence on their index date using data in the Medicare beneficiary summary file or the population file in MarketScan. Geographic regions of residence to be analyzed include:   - West – includes the following states: WA, OR, ID, WY, NV, UT, CO, AZ, and NM - Midwest – includes the following states: ND, MN, SD, NE, IA, KS, MO, WI, MI, IL, IN, and OH - Northeast – includes the following states: NY, PA, NK, ME, NH, V, MA, and CT - South – includes the following states: OK, AR, TX, LA, FL, GA, SC, NC, VA, TN, KY, MS. AL, WV, MD, and DE |
| Area-level income (among Medicare patients only) | Median income by zip code were obtained from US census data and merged to the Medicare claims using each beneficiary’s residential zip code. Area-level income was grouped into four categories: <$25,000, $25,000 - $49,999, $50,000 - $74,999, and ≥$75,000. |
| Use of insulin | ≥1 pharmacy claim for insulin within 365 days prior to the index date, inclusive |
| History of heart failure | Any of the following using all available claims prior to the index date, inclusive:   - ≥1 hospitalization with a discharge diagnosis code of heart failure (ICD-9-CM diagnosis code of 402.01, 402.11, 402.91, 404.01, 404.03, 404.11, 404.13, 404.91, 404.93, or 428.x) in any discharge diagnosis position or ICD10 of 'I110', 'I130', 'I132', 'I501', 'I5020', 'I5021', 'I5022', 'I5023', 'I5030', 'I5031', 'I5032', 'I5033', 'I5040', 'I5041', 'I5042', 'I5043', 'I509') - ≥2 physician evaluation and management visits with a diagnosis code of heart failure (ICD-9-CM diagnosis code of 402.01, 402.11, 402.91, 404.01, 404.03, 404.11, 404.13, 404.91, 404.93, or 428.x) or ICD10 of 'I110', 'I130', 'I132', 'I501', 'I5020', 'I5021', 'I5022', 'I5023', 'I5030', 'I5031', 'I5032', 'I5033', 'I5040', 'I5041', 'I5042', 'I5043', 'I509') in any position on separate days |
| Prior MI hospitalization | All available claims prior to the admission for the MI hospitalization related to the index date:  ≥1 hospitalization with a diagnosis code of MI (ICD-9-CM diagnosis code of 410.xx or 412.xx or an ICD-10 code I21.xx I22.xx) in any discharge diagnosis position |
| Prior coronary artery bypass surgery or percutaneous coronary intervention outside of an MI event | All available claims prior to the index date, inclusive:  ≥1 hospitalization or physician visit with a procedure code for coronary revascularization (ICD-9-CM procedure codes 00.66, 36.0, 36.01-36.19, 36.2 or CPT codes 33510-33519, 33521-33523, 33530, 33533-33536, 92980-92982, 92984, 92995, 92996) or (ICD-10 procedure code starting with any of the following 4 digits: 0210, 0211, 0212, 0213, 0270, 0271, 0272, 0273, 02C0, 02C1, 02C2, 02C3, 3E07).  Exclude revascularizations on or within 60 days after an MI event. |
| Coronary artery bypass surgery or percutaneous coronary intervention during the MI hospitalization related to the index date. | During hospitalization, inclusive:  ≥1 hospitalization or physician visit with a procedure code for coronary revascularization (ICD-9-CM procedure codes 00.66, 36.0, 36.01-36.19, 36.2, ICD-9-CM diagnosis codes V45.81 or V45.82, or CPT codes 33510-33519, 33521-33523, 33530, 33533-33536, 92980-92982, 92984, 92995, 92996) or (ICD-10 procedure code starting with any of the following 4 digits: 0210, 0211, 0212, 0213, 0270, 0271, 0272, 0273, 02C0, 02C1, 02C2, 02C3, 3E07) during the index MI hospitalization (on or prior to the index date). |
| Cardiologist care | ≥1 physician evaluation and management visit with a specialty code 06 in Medicare or a provider type 250 in MarketScan within 365 days prior to the index date, inclusive. |
| Endocrinologist care | ≥1 physician evaluation and management visit with a specialty code 46 in Medicare or a provider type 270 in MarketScan within 365 days prior to the index date, inclusive. |
| Depression | Any of the following within 365 days prior to each patient’s index date, inclusive:   - ≥1 inpatient claim with ICD-9 diagnosis code (any position) of 296.20-296.26, 296.30-296.36, 296.51-296.56, 296.60-296.66, 296.89, 298.0, 300.4, 309.1 or 311, or ICD10 diagnosis code of 'F329', 'F320', 'F321','F322', 'F323', 'F324', 'F325', 'F339', 'F330', 'F331', 'F332', 'F333', 'F3341', 'F3342', 'F3131', 'F3132', 'F314', 'F315', 'F3175', 'F3176', 'F3160', 'F3161','F3162', 'F3163', 'F3164', 'F3177', 'F3178', 'F3181', 'F323', 'F333', 'F341', 'F4321', 'F329'. - ≥1 evaluation and management outpatient claim with ICD-9 diagnosis code (any position) of 296.20-296.26, 296.30-296.36, 296.51-296.56, 296.60-296.66, 296.89, 298.0, 300.4, 309.1 or 311. - ≥2 pharmacy claims for amitriptyline, amoxapine, bupropion, citalopram, clomipramine, desipramine, desvenlafaxine, doxepin, duloxetine, escitalopram, fluoxetine, fluvoxamine, imipramine, isocarboxazid, levomilnacipran, maprotiline, milnacipran, mirtazapine, nefazodone, nortriptyline, paroxetine, perphenazine, phenelzine, protriptyline, selegiline, sertraline, tranylcypromine, trazodone, trimipramine or venlafaxine in a separate day. |
| Smoking | Any of the following within 365 days prior to each patient’s index date, inclusive:   - 1 hospitalization with a discharge diagnosis code of tobacco use (ICD-9 CM diagnosis code of 305.1, 649.0x, 989.84, or V15.82 or ICD-10 CM diagnosis code of F17.200, F17.201, F17.210, F17.211, F17.220, F17.221, F17.290, F17.291, O99.330, O99.331, O99.332, O99.333, O99.334, O99.335, T65.211A, T65.212A, T65.213A, T65.214A, T65.221A, T65.222A, T65.223A, T65.224A, T65.291A, T65.292A, T65.293A, T65.294A, or Z87.891) in any discharge position - ≥1 physician evaluation and management visit with a diagnosis code of tobacco use (ICD-9-CM diagnosis code of 305.1, 649.0x, 989.84, or V15.82 or ICD-10 CM diagnosis code of F17.200, F17.201, F17.210, F17.211, F17.220, F17.221, F17.290, F17.291, O99.330, O99.331, O99.332, O99.333, O99.334, O99.335, T65.211A, T65.212A, T65.213A, T65.214A, T65.221A, T65.222A, T65.223A, T65.224A, T65.291A, T65.292A, T65.293A, T65.294A, or Z87.891) in any discharge position - ≥1 physician evaluation and management visit of tobacco use with a CPT code of 99406, 99407, G0436, G0437, G9016, S9453, S4995, G9276, G9458, 1034F, 4004F, 4001F - ≥1 pharmacy claim for nicotine or varenicline |
| Hypertension | All available claims prior to the index date, inclusive:   - ≥1 inpatient claim with an ICD-9 discharge diagnosis code of 401.x, 403.0x, 403.1x, 403.9x or an ICD-10 discharge diagnosis code of I10, I12.0, I12.9 in any discharge diagnosis position. - ≥2 outpatient claims with an ICD-9 diagnosis code of 401.x, 403.0x, 403.1x, 403.9x or an ICD-10 diagnosis code of I10, I12.0, and I12.9 in any position at least 30 days apart. |
| Statin use and intensity | No statin use was defined by having no prescription fill for any statin dose and type within 365 days prior to the hospital admission date.  Use of low/moderate-intensity statin was defined by ≥1 prescription fill for a low/moderate-intensity statin with no high-intensity statin fills within 365 days prior to the hospital admission date.  Use of high-intensity statin was defined by ≥1 prescription fill for Atorvastatin 40 or 80 mg, or Rosuvastatin 20 or 40 mg within 365 days prior to the hospital admission date. |
| Ezetimibe use | ≥1 prescription fill for ezetimibe within 365 days prior to the hospital admission date. |
| Antihypertensive medication use | ≥1 prescription fill for any thiazides, angiotensin-converting-enzyme inhibitors, angiotensin II receptor blockers, calcium channel blockers, diuretics, beta blockers, direct renin inhibitors, alpha-1 blockers, central alpha1-agonists or direct vasodilators 365 days prior to or on the index date, inclusive. |
| CPT: Current Procedure Terminology; HCPSC: Healthcare Common Procedure Coding System; ICD-9-CM: International Classification of Diseases, Ninth Revision, Clinical Modification; ICD-10-CM: International Classification of Diseases, Tenth Revision, Clinical Modification; MI: myocardial infarction.  In Medicare, physician evaluation and management visits were defined by a CPT code 99024, 99058, 99429, 99499, 99201-99215, 99241-99245, 99271-99275, 99301-99337, 99341-99355, 99385-99387, or 99395-99404, or 99281-99285. In MarketScan, physician evaluation and management visits were defined by an encounter type “AV”, “NH” or “ED”. | |

Supplemental Table 4. Definitions of outcomes

| **Outcome** | **Definition** |
| --- | --- |
| CVD events | Defined as the first occurrence of either a recurrent MI, CHD, stroke, or peripheral artery disease event as defined below following the index date. |
| Recurrent MI | Overnight hospitalization with a discharge diagnosis code for MI (i.e., an ICD-9 code 410.xx, except 410.x2, which represent a subsequent episode of care or an ICD-10 of code I21.xx) in any discharge diagnosis position |
| Coronary heart disease event | Defined by a recurrent MI (as described above) or a coronary revascularization procedure. A coronary revascularization procedure was defined by an inpatient or outpatient procedure with a current procedure terminology (CPT) code for coronary revascularization (see below), an ICD-9 procedure code of 00.66, 36.0, 36.01-36.19, 36.2, or an ICD-10 procedure code starting with any of the following 4 digits: 0210, 0211, 0212, 0213, 0270, 0271, 0272, 0273, 02C0, 02C1, 02C2, 02C3, 3E07. Some CPT codes for coronary revascularization change by calendar year. CPT codes for coronary revascularization in any calendar year include 33510-33519, 33521-33523, 33530, 33533-33536. CPT codes for coronary revascularization through 2012 include 92980-92982, 92984, 92995, and 92996. CPT codes for coronary revascularization in 2013 and after including 92920, 92921, 92924, 92925, 92928, 92929, 92933, 92934, 92937, 92938, 92941, 92943, and 92944. Coronary revascularizations in the 60 days following an MI hospitalization may be elective and not represent a new coronary event. Therefore, coronary revascularizations in the 60 days following MI were included if these were linked to a primary discharge diagnosis for non-elective CHD-related hospitalization (arrhythmia [ICD-9 diagnosis code of 427.xx, except 427.5 or ICD-10 diagnosis code of I47.1, I47.2, I47.9, I48.91, I48.92, I49.01, I49.02, I49.1, I49.3, I49.40, I49.49, I49.5, I49.8, I49.9, R00.1], cardiac arrest [ICD-9 diagnosis code of 427.5 or ICD-10 diagnosis code of I46.9], heart failure [ICD-9 diagnosis code of 402.01, 402.11, 402.91, 404.01, 404.03, 404.11, 404.13, 404.91, 404.93, 428.x or ICD-10 diagnosis code of I11.0, I13.0, I13.2, I50.1, I50.20, I50.21, I50.22, I50.23, I50.30, I50.31, I50.32, I50.33, I50.40, I50.41, I50.42, I50.43, I50.9], and unstable angina [ICD-9 diagnosis code of 411.xx or ICD-10 diagnosis code of I20.0, I24.0, I24.1, I24.8]). |
| Stroke event | An inpatient claim with a discharge diagnosis code for ischemic or hemorrhagic stroke (i.e., ICD-9 codes 430.xx, 431.xx, 433.x1, 434.x1 or 436.x, or ICD-10 codes of I60.xx, I61.xx, I63.xx and I67.89) as recorded in the primary discharge diagnosis position. |
| Peripheral artery disease event | The earliest of the following events:   - An overnight inpatient claim with a discharge diagnosis code for acute limb ischemia in the primary discharge diagnosis position. - An overnight inpatient claim with a procedure code for embolectomy, thrombectomy or peripheral surgical revascularization in any position. - An overnight inpatient claim with a procedure code for thrombolysis in the absence of a discharge diagnosis code for acute myocardial infarction, ischemic stroke or pulmonary embolism in any position. - An overnight inpatient claim with a procedure code for lower extremity amputation above the ankle in any position, in the absence of a discharge diagnosis code for traumatic amputation of a leg on the same hospitalization. Amputations were counted as an event only if the patient had ≥1 inpatient or outpatient claim with a diagnosis code for peripheral artery disease in any position prior to or on the date of the amputation.   List of codes:  ICD9 diagnosis codes for acute limb ischemia: 444.0, 444.01, 444.09, 444.22, 444.81.  ICD10 diagnosis codes for acute limb ischemia: I74.01, I74.09, I74.3, I74.5.  CPT procedure codes for embolectomy or thrombectomy: 34201, 34203.  ICD9 procedure codes for peripheral surgical revascularization: 38.08, 38.16, 38.18, 38.38, 38.48, 38.68, 38.88, 39.25, 39.29.  ICD10 procedure codes for peripheral surgical revascularization: 0312096, 0312097, 0312098, 0312099, 031209B, 031209C, 03120A6, 03120A7, 03120A8, 03120A9, 03120AB, 03120AC, 03120J6, 03120J7, 03120J8, 03120J9, 03120JB, 03120JC, 03120K6, 03120K7, 03120K8, 03120K9, 03120KB, 03120KC, 03120Z6, 03120Z7, 03120Z8, 03120Z9, 03120ZB, 03120ZC, 031309B, 031309C, 03130A6, 03130A7, 03130A8, 03130A9, 03130AB, 03130AC, 03130J6, 03130J7, 03130J8, 03130J9, 03130JB, 03130JC, 03130K6, 03130K7, 03130K8, 03130K9, 03130KB, 03130KC, 03130Z6, 03130Z7, 03130Z8, 03130Z9, 03130ZB, 03130ZC, 0314096, 0314097, 0314098, 0314099, 031409B, 031409C, 03140A6, 03140A7, 03140A8, 03140A9, 03140AB, 03140AC, 03140J6, 03140J7, 03140J8, 03140J9, 03140JB, 03140JC, 03140K6, 03140K7, 03140K8, 03140K9, 03140KB, 03140KC, 03140Z6, 03140Z7, 03140Z8, 03140Z9, 03140ZB, 03140ZC, 0315096, 0315097, 0315098, 0315099, 031509B, 031509C, 03150A6, 03150A7, 03150A8, 03150A9, 03150AB, 03150AC, 03150J6, 03150J7, 03150J8, 03150J9, 03150JB, 03150JC, 03150K6, 03150K7, 03150K8, 03150K9, 03150KB, 03150KC, 03150Z6, 03150Z7, 03150Z8, 03150Z9, 03150ZB, 03150ZC, 0316096, 0316097, 0316098, 0316099, 031609B, 031609C, 03160A6, 03160A7, 03160A8, 03160A9, 03160AB, 03160AC, 03160J6, 03160J7, 03160J8, 03160J9, 03160JB, 03160JC, 03160K6, 03160K7, 03160K8, 03160K9, 03160KB, 03160KC, 03160Z6, 03160Z7, 03160Z8, 03160Z9, 03160ZB, 03160ZC, 0410096, 0410097, 0410098, 0410099, 041009B, 041009C, 041009D, 041009F, 041009G, 041009H, 041009J, 041009K, 041009Q, 041009R, 04100A6, 04100A7, 04100A8, 04100A9, 04100AB, 04100AC, 04100AD, 04100AF, 04100AG, 04100AH, 04100AJ, 04100AK, 04100AQ, 04100AR, 04100J6, 04100J7, 04100J8, 04100J9, 04100JB, 04100JC, 04100JD, 04100JF, 04100JG, 04100JH, 04100JJ, 04100JK, 04100JQ, 04100JR, 04100K6, 04100K7, 04100K8, 04100K9, 04100KB, 04100KC, 04100KD, 04100KF, 04100KG, 04100KH, 04100KJ, 04100KK, 04100KQ, 04100KR, 04100Z6, 04100Z7, 04100Z8, 04100Z9, 04100ZB, 04100ZC, 04100ZD, 04100ZF, 04100ZG, 04100ZH, 04100ZJ, 04100ZK, 04100ZQ, 04100ZR, 0410496, 0410497, 0410498, 0410499, 041049B, 041049C, 041049D, 041049F, 041049G, 041049H, 041049J, 041049K, 041049Q, 041049R, 04104A6, 04104A7, 04104A8, 04104A9, 04104AB, 04104AC, 04104AD, 04104AF, 04104AG, 04104AH, 04104AJ, 04104AK, 04104AQ, 04104AR, 04104J6, 04104J7, 04104J8, 04104J9, 04104JB, 04104JC, 04104JD, 04104JF, 04104JG, 04104JH, 04104JJ, 04104JK, 04104JQ, 04104JR, 04104K6, 04104K7, 04104K8, 04104K9, 04104KB, 04104KC, 04104KD, 04104KF, 04104KG, 04104KH, 04104KJ, 04104KK, 04104KQ, 04104KR, 04104Z6, 04104Z7, 04104Z8, 04104Z9, 04104ZB, 04104ZC, 04104ZD, 04104ZF, 04104ZG, 04104ZH, 04104ZJ, 04104ZK, 04104ZQ, 04104ZR, 041C096, 041C097, 041C098, 041C099, 041C09B, 041C09C, 041C09D, 041C09F, 041C09G, 041C09H, 041C09J, 041C09K, 041C09Q, 041C0AH, 041C0AJ, 041C0AK, 041C0J6, 041C0J7, 041C0J8, 041C0J9, 041C0JB, 041C0JC, 041C0JD, 041C0JF, 041C0JG, 041C0JH, 041C0JJ, 041C0JK, 041C0JQ, 041C0K6, 041C0K7, 041C0K8, 041C0K9, 041C0KB, 041C0KC, 041C0KD, 041C0KF, 041C0KG, 041C0KH, 041C0KJ, 041C0KK, 041C0Z6, 041C0Z7, 041C0Z8, 041C0Z9, 041C0ZB, 041C0ZC, 041C0ZD, 041C0ZF, 041C0ZG, 041C0ZH, 041C0ZJ, 041C0ZK, 041C0ZQ, 041C496, 041C497, 041C498, 041C499, 041C49B, 041C49C, 041C49D, 041C49F, 041C49G, 041C49H, 041C49J, 041C49K, 041C49Q, 041C4A6, 041C4A7, 041C4A8, 041C4A9, 041C4AB, 041C4AC, 041C4AD, 041C4AF, 041C4AG, 041C4AH, 041C4AJ, 041C4AK, 041C4J6, 041C4J7, 041C4J8, 041C4J9, 041C4JB, 041C4JC, 041C4JD, 041C4JF, 041C4JG, 041C4JH, 041C4JJ, 041C4JK, 041C4JQ, 041C4K6, 041C4K7, 041C4K8, 041C4K9, 041C4KB, 041C4KC, 041C4KD, 041C4KF, 041C4KG, 041C4KH, 041C4KJ, 041C4KK, 041C4KQ, 041C4Z6, 041C4Z7, 041C4Z8, 041C4Z9, 041C4ZB, 041C4ZC, 041C4ZD, 041C4ZF, 041C4ZG, 041C4ZH, 041C4ZJ, 041C4ZK, 041C4ZQ, 041D098, 041D099, 041D09B, 041D09C, 041D09D, 041D09F, 041D09G, 041D09H, 041D09J, 041D09K, 041D09Q, 041D0A6, 041D0A7, 041D0A8, 041D0A9, 041D0AB, 041D0AC, 041D0AD, 041D0AF, 041D0AG, 041D0AH, 041D0AJ, 041D0AK, 041D0AQ, 041D0J6, 041D0J7, 041D0J8, 041D0J9, 041D0JB, 041D0JC, 041D0JD, 041D0JF, 041D0JG, 041D0JH, 041D0JJ, 041D0JK, 041D0JQ, 041D0K6, 041D0K7, 041D0K8, 041D0K9, 041D0KB, 041D0KC, 041D0KD, 041D0KF, 041D0KG, 041D0KH, 041D0KJ, 041D0KK, 041D0KQ, 041D0Z6, 041D0Z7, 041D0Z8, 041D0Z9, 041D0ZB, 041D0ZC, 041D0ZD, 041D0ZF, 041D0ZG, 041D0ZH, 041D0ZJ, 041D0ZK, 041D0ZQ, 041D496, 041D497, 041D498, 041D499, 041D49B, 041D49C, 041D49D, 041D49F, 041D49G, 041D49H, 041D49J, 041D49K, 041D49Q, 041D4A6, 041D4A7, 041D4A8, 041D4A9, 041D4AB, 041D4AC, 041D4AD, 041D4AF, 041D4AG, 041D4AH, 041D4AJ, 041D4AK, 041D4AQ, 041D4J6, 041D4J7, 041D4J8, 041D4J9, 041D4JB, 041D4JC, 041D4JD, 041D4JF, 041D4JG, 041D4JH, 041D4JJ, 041D4JK, 041D4JQ, 041D4K7, 041D4K8, 041D4K9, 041D4KB, 041D4KC, 041D4KD, 041D4KF, 041D4KG, 041D4KH, 041D4KJ, 041D4KK, 041D4KQ, 041D4Z6, 041D4Z7, 041D4Z8, 041D4Z9, 041D4ZB, 041D4ZC, 041D4ZD, 041D4ZF, 041D4ZG, 041D4ZH, 041D4ZJ, 041D4ZK, 041D4ZQ, 041D4ZR, 041E099, 041E09B, 041E09C, 041E09D, 041E09F, 041E09G, 041E09H, 041E09J, 041E09K, 041E0A9, 041E0AB, 041E0AC, 041E0AD, 041E0AF, 041E0AG, 041E0AH, 041E0AJ, 041E0AK, 041E0AP, 041E0AQ, 041E0J9, 041E0JB, 041E0JC, 041E0JD, 041E0JF, 041E0JG, 041E0JH, 041E0JJ, 041E0JK, 041E0JP, 041E0JQ, 041E0K9, 041E0KB, 041E0KC, 041E0KD, 041E0KF, 041E0KG, 041E0KH, 041E0KJ, 041E0KK, 041E0KP, 041E0KQ, 041E0Z9, 041E0ZB, 041E0ZC, 041E0ZD, 041E0ZF, 041E0ZG, 041E0ZH, 041E0ZJ, 041E0ZK, 041E0ZP, 041E0ZQ, 041E499, 041E49B, 041E49C, 041E49D, 041E49F, 041E49G, 041E49H, 041E49J, 041E49K, 041E49P, 041E49Q, 041E4A9, 041E4AB, 041E4AC, 041E4AD, 041E4AF, 041E4AG, 041E4AH, 041E4AJ, 041E4AK, 041E4AP, 041E4AQ, 041E4J9, 041E4JB, 041E4JC, 041E4JD, 041E4JF, 041E4JG, 041E4JH, 041E4JJ, 041E4JK, 041E4JP, 041E4JQ, 041E4K9, 041E4KB, 041E4KC, 041E4KD, 041E4KF, 041E4KG, 041E4KH, 041E4KJ, 041E4KK, 041E4KP, 041E4KQ, 041E4Z9, 041E4ZB, 041E4ZC, 041E4ZD, 041E4ZF, 041E4ZG, 041E4ZH, 041E4ZJ, 041E4ZK, 041E4ZP, 041E4ZQ, 041F099, 041F09B, 041F09C, 041F09D, 041F09F, 041F09G, 041F09H, 041F09J, 041F09K, 041F09P, 041F09Q, 041F0A9, 041F0AB, 041F0AC, 041F0AD, 041F0AF, 041F0AG, 041F0AH, 041F0AJ, 041F0AK, 041F0AP, 041F0AQ, 041F0J9, 041F0JB, 041F0JC, 041F0JD, 041F0JF, 041F0JG, 041F0JH, 041F0JJ, 041F0JK, 041F0JP, 041F0JQ, 041F0K9, 041F0KB, 041F0KC, 041F0KD, 041F0KF, 041F0KG, 041F0KH, 041F0KJ, 041F0KK, 041F0KP, 041F0KQ, 041F0Z9, 041F0ZB, 041F0ZC, 041F0ZD, 041F0ZF, 041F0ZG, 041F0ZH, 041F0ZJ, 041F0ZK, 041F0ZP, 041F0ZQ, 041F499, 041F49B, 041F49C, 041F49D, 041F49F, 041F49G, 041F49H, 041F49J, 041F49K, 041F49P, 041F49Q, 041F4A9, 041F4AB, 041F4AC, 041F4AD, 041F4AF, 041F4AG, 041F4AH, 041F4AJ, 041F4AK, 041F4AP, 041F4AQ, 041F4J9, 041F4JB, 041F4JC, 041F4JD, 041F4JF, 041F4JG, 041F4JH, 041F4JJ, 041F4JK, 041F4JP, 041F4JQ, 041F4K9, 041F4KB, 041F4KC, 041F4KD, 041F4KF, 041F4KG, 041F4KH, 041F4KJ, 041F4KK, 041F4KP, 041F4KQ, 041F4Z9, 041F4ZB, 041F4ZC, 041F4ZD, 041F4ZF, 041F4ZG, 041F4ZH, 041F4ZJ, 041F4ZK, 041F4ZP, 041F4ZQ, 041H099, 041H09B, 041H09C, 041H09D, 041H09F, 041H09G, 041H09H, 041H09J, 041H09K, 041H09P, 041H09Q, 041H0A9, 041H0AB, 041H0AC, 041H0AD, 041H0AF, 041H0AG, 041H0AH, 041H0AJ, 041H0AK, 041H0AP, 041H0AQ, 041H0J9, 041H0JB, 041H0JC, 041H0JD, 041H0JF, 041H0JG, 041H0JH, 041H0JJ, 041H0JK, 041H0JP, 041H0JQ, 041H0K9, 041H0KB, 041H0KC, 041H0KD, 041H0KF, 041H0KG, 041H0KH, 041H0KJ, 041H0KK, 041H0KP, 041H0KQ, 041H0Z9, 041H0ZB, 041H0ZC, 041H0ZD, 041H0ZF, 041H0ZG, 041H0ZH, 041H0ZJ, 041H0ZK, 041H0ZP, 041H0ZQ, 041H499, 041H49B, 041H49C, 041H49D, 041H49F, 041H49G, 041H49H, 041H49J, 041H49K, 041H49P, 041H49Q, 041H4A9, 041H4AB, 041H4AC, 041H4AD, 041H4AF, 041H4AG, 041H4AH, 041H4AJ, 041H4AK, 041H4AP, 041H4AQ, 041H4J9, 041H4JB, 041H4JC, 041H4JD, 041H4JF, 041H4JG, 041H4JH, 041H4JJ, 041H4JK, 041H4JP, 041H4JQ, 041H4K9, 041H4KB, 041H4KC, 041H4KD, 041H4KF, 041H4KG, 041H4KH, 041H4KJ, 041H4KK, 041H4KP, 041H4KQ, 041H4Z9, 041H4ZB, 041H4ZC, 041H4ZD, 041H4ZF, 041H4ZG, 041H4ZH, 041H4ZJ, 041H4ZK, 041H4ZP, 041H4ZQ, 041J099, 041J09B, 041J09C, 041J09D, 041J09F, 041J09G, 041J09H, 041J09J, 041J09K, 041J09P, 041J09Q, 041J0A9, 041J0AB, 041J0AC, 041J0AD, 041J0AF, 041J0AG, 041J0AH, 041J0AJ, 041J0AK, 041J0AP, 041J0AQ, 041J0J9, 041J0JB, 041J0JC, 041J0JD, 041J0JF, 041J0JG, 041J0JH, 041J0JJ, 041J0JK, 041J0JP, 041J0JQ, 041J0K9, 041J0KB, 041J0KC, 041J0KD, 041J0KF, 041J0KG, 041J0KH, 041J0KJ, 041J0KK, 041J0KP, 041J0KQ, 041J0Z9, 041J0ZB, 041J0ZC, 041J0ZD, 041J0ZF, 041J0ZG, 041J0ZH, 041J0ZJ, 041J0ZK, 041J0ZP, 041J0ZQ, 041J499, 041J49B, 041J49C, 041J49D, 041J49F, 041J49G, 041J49H, 041J49J, 041J49K, 041J49P, 041J49Q, 041J4A9, 041J4AB, 041J4AC, 041J4AD, 041J4AF, 041J4AG, 041J4AH, 041J4AJ, 041J4AK, 041J4AP, 041J4AQ, 041J4J9, 041J4JB, 041J4JC, 041J4JD, 041J4JF, 041J4JH, 041J4JJ, 041J4JK, 041J4JP, 041J4JQ, 041J4K9, 041J4KB, 041J4KC, 041J4KD, 041J4KF, 041J4KG, 041J4KH, 041J4KJ, 041J4KK, 041J4KP, 041J4KQ, 041J4Z9, 041J4ZB, 041J4ZC, 041J4ZD, 041J4ZF, 041J4ZG, 041J4ZH, 041J4ZJ, 041J4ZK, 041J4ZP, 041J4ZQ, 041K09H, 041K09J, 041K09K, 041K09L, 041K09M, 041K09N, 041K09P, 041K09Q, 041K0AH, 041K0AJ, 041K0AK, 041K0AL, 041K0AM, 041K0AN, 041K0AP, 041K0AQ, 041K0JH, 041K0JJ, 041K0JK, 041K0JL, 041K0JM, 041K0JN, 041K0JP, 041K0JQ, 041K0KH, 041K0KJ, 041K0KK, 041K0KL, 041K0KM, 041K0KN, 041K0KP, 041K0KQ, 041K0ZH, 041K0ZJ, 041K0ZK, 041K0ZL, 041K0ZM, 041K0ZN, 041K0ZP, 041K0ZQ, 041K49H, 041K49J, 041K49K, 041K49L, 041K49M, 041K49N, 041K49P, 041K49Q, 041K4AH, 041K4AJ, 041K4AK, 041K4AL, 041K4AM, 041K4AN, 041K4AP, 041K4AQ, 041K4JH, 041K4JJ, 041K4JK, 041K4JL, 041K4JM, 041K4JN, 041K4JP, 041K4JQ, 041K4KH, 041K4KJ, 041K4KK, 041K4KL, 041K4KM, 041K4KN, 041K4KP, 041K4KQ, 041K4ZH, 041K4ZJ, 041K4ZK, 041K4ZL, 041K4ZM, 041K4ZN, 041K4ZP, 041K4ZQ, 041L09H, 041L09J, 041L09K, 041L09L, 041L09M, 041L09N, 041L09P, 041L09Q, 041L0AH, 041L0AJ, 041L0AK, 041L0AL, 041L0AM, 041L0AN, 041L0AP, 041L0AQ, 041L0JH, 041L0JJ, 041L0JK, 041L0JL, 041L0JM, 041L0JN, 041L0JP, 041L0JQ, 041L0KH, 041L0KJ, 041L0KK, 041L0KL, 041L0KM, 041L0KN, 041L0KP, 041L0KQ, 041L0ZH, 041L0ZJ, 041L0ZK, 041L0ZL, 041L0ZM, 041L0ZN, 041L0ZP, 041L0ZQ, 041L49H, 041L49J, 041L49K, 041L49L, 041L49M, 041L49N, 041L49P, 041L49Q, 041L4AH, 041L4AJ, 041L4AK, 041L4AL, 041L4AM, 041L4AN, 041L4AP, 041L4AQ, 041L4JH, 041L4JJ, 041L4JK, 041L4JL, 041L4JM, 041L4JN, 041L4JP, 041L4JQ, 041L4KH, 041L4KJ, 041L4KK, 041L4KL, 041L4KM, 041L4KN, 041L4KP, 041L4KQ, 041L4ZH, 041L4ZJ, 041L4ZK, 041L4ZL, 041L4ZM, 041L4ZN, 041L4ZP, 041L4ZQ, 041M09L, 041M09M, 041M09P, 041M09Q, 041M0AL, 041M0AM, 041M0AP, 041M0AQ, 041M0JL, 041M0JM, 041M0JP, 041M0JQ, 041M0KL, 041M0KM, 041M0KP, 041M0KQ, 041M0ZL, 041M0ZM, 041M0ZP, 041M0ZQ, 041M49L, 041M49M, 041M49P, 041M49Q, 041M4AL, 041M4AM, 041M4AP, 041M4AQ, 041M4JL, 041M4JM, 041M4JP, 041M4JQ, 041M4KL, 041M4KM, 041M4KP, 041M4KQ, 041M4ZL, 041M4ZM, 041M4ZP, 041M4ZQ, 041N09L, 041N09M, 041N09P, 041N09Q, 041N0AL, 041N0AM, 041N0AP, 041N0AQ, 041N0JL, 041N0JM, 041N0JP, 041N0JQ, 041N0KL, 041N0KM, 041N0KP, 041N0KQ, 041N0ZL, 041N0ZM, 041N0ZP, 041N0ZQ, 041N49L, 041N49M, 041N49P, 041N49Q, 041N4AL, 041N4AM, 041N4AP, 041N4AQ, 041N4JL, 041N4JM, 041N4JP, 041N4JQ, 041N4KL, 041N4KM, 041N4KP, 041N4KQ, 041N4ZL, 041N4ZM, 041N4ZP, 041N4ZQ, 041T09P, 041T09Q, 041T0AP, 041T0AQ, 041T0JP, 041T0JQ, 041T0KP, 041T0KQ, 041T0ZP, 041T0ZQ, 041T49P, 041T49Q, 041T4AP, 041T4AQ, 041T4JP, 041T4JQ, 041T4KP, 041T4KQ, 041T4ZP, 041T4ZQ, 041U09P, 041U09Q, 041U0AP, 041U0AQ, 041U0JP, 041U0JQ, 041U0KP, 041U0KQ, 041U0ZP, 041U0ZQ, 041U49P, 041U49Q, 041U4AP, 041U4AQ, 041U4JP, 041U4JQ, 041U4KP, 041U4KQ, 041U4ZP, 041U4ZQ, 041V09P, 041V09Q, 041V0AP, 041V0AQ, 041V0JP, 041V0JQ, 041V0KP, 041V0KQ, 041V0ZP, 041V0ZQ, 041V49P, 041V49Q, 041V4AP, 041V4AQ, 041V4JP, 041V4JQ, 041V4KP, 041V4KQ, 041V4ZP, 041V4ZQ, 041W09P, 041W09Q, 041W0AP, 041W0AQ, 041W0JP, 041W0JQ, 041W0KP, 041W0KQ, 041W0ZP, 041W0ZQ, 041W49P, 041W49Q, 041W4AP, 041W4AQ, 041W4JP, 041W4JQ, 041W4KP, 041W4KQ, 041W4ZP, 041W4ZQ, 04BK0ZZ, 04BK3ZZ, 04BK4ZZ, 04BL0ZZ, 04BL3ZZ, 04BL4ZZ, 04BM0ZZ, 04BM3ZZ, 04BM4ZZ, 04BN0ZZ, 04BN3ZZ, 04BN4ZZ, 04BP0ZZ, 04BP3ZZ, 04BP4ZZ, 04BQ0ZZ, 04BQ3ZZ, 04BQ4ZZ, 04BR0ZZ, 04BR3ZZ, 04BR4ZZ, 04BS0ZZ, 04BS3ZZ, 04BS4ZZ, 04BT0ZZ, 04BT3ZZ, 04BT4ZZ, 04BU0ZZ, 04BU3ZZ, 04BU4ZZ, 04BV0ZZ, 04BV3ZZ, 04BV4ZZ, 04BW0ZZ, 04BW3ZZ, 04BW4ZZ, 04BY0ZZ, 04BY3ZZ, 04BY4ZZ, 04CC0ZZ, 04CC3ZZ, 04CC4ZZ, 04CD0ZZ, 04CD3ZZ, 04CD4ZZ, 04CE0ZZ, 04CE3ZZ, 04CE4ZZ, 04CF0ZZ, 04CF3ZZ, 04CF4ZZ, 04CH0ZZ, 04CH3ZZ, 04CH4ZZ, 04CJ0ZZ, 04CJ3ZZ, 04CJ4ZZ, 04CK0Z6, 04CK0ZZ, 04CK3ZZ, 04CK4Z6, 04CK4ZZ, 04CL0Z6, 04CL0ZZ, 04CL3ZZ, 04CL4Z6, 04CL4ZZ, 04CM0Z6, 04CM0ZZ, 04CM3ZZ, 04CM4Z6, 04CM4ZZ, 04CN0Z6, 04CN0ZZ, 04CN3ZZ, 04CN4Z6, 04CN4ZZ, 04CP0Z6, 04CP0ZZ, 04CP3ZZ, 04CP4Z6, 04CP4ZZ, 04CQ0Z6, 04CQ0ZZ, 04CQ3ZZ, 04CQ4Z6, 04CQ4ZZ, 04CR0Z6, 04CR0ZZ, 04CR3ZZ, 04CR4Z6, 04CR4ZZ, 04CS0Z6, 04CS0ZZ, 04CS3ZZ, 04CS4Z6, 04CS4ZZ, 04CT0Z6, 04CT0ZZ, 04CT3ZZ, 04CT4Z6, 04CT4ZZ, 04CU0Z6, 04CU0ZZ, 04CU3ZZ, 04CU4Z6, 04CU4ZZ, 04CV0Z6, 04CV0ZZ, 04CV3ZZ, 04CV4Z6, 04CV4ZZ, 04CW0Z6, 04CW0ZZ, 04CW3ZZ, 04CW4Z6, 04CW4ZZ, 04CY0Z6, 04CY0ZZ, 04CY3ZZ, 04CY4Z6, 04CY4ZZ, 04HY02Z, 04HY42Z, 04PY0YZ, 04PY3YZ, 04PY4YZ, 04RK07Z, 04RK0JZ, 04RK0KZ, 04RK47Z, 04RK4JZ, 04RK4KZ, 04RL07Z, 04RL0JZ, 04RL0KZ, 04RL47Z, 04RL4JZ, 04RL4KZ, 04RM07Z, 04RM0JZ, 04RM0KZ, 04RM47Z, 04RM4JZ, 04RM4KZ, 04RN07Z, 04RN0JZ, 04RN0KZ, 04RN47Z, 04RN4JZ, 04RN4KZ, 04RP07Z, 04RP0JZ, 04RP0KZ, 04RP47Z, 04RP4JZ, 04RP4KZ, 04RQ07Z, 04RQ0JZ, 04RQ0KZ, 04RQ47Z, 04RQ4JZ, 04RQ4KZ, 04RR07Z, 04RR0JZ, 04RR0KZ, 04RR47Z, 04RR4JZ, 04RR4KZ, 04RS07Z, 04RS0JZ, 04RS0KZ, 04RS47Z, 04RS4JZ, 04RS4KZ, 04RT07Z, 04RT0JZ, 04RT0KZ, 04RT47Z, 04RT4JZ, 04RT4KZ, 04RU07Z, 04RU0JZ, 04RU0KZ, 04RU47Z, 04RU4JZ, 04RU4KZ, 04RV07Z, 04RV0JZ, 04RV0KZ, 04RV47Z, 04RV4JZ, 04RV4KZ, 04RW07Z, 04RW0JZ, 04RW0KZ, 04RW47Z, 04RW4JZ, 04RW4KZ, 04RY07Z, 04RY0JZ, 04RY0KZ, 04RY47Z, 04RY4JZ, 04RY4KZ, 04WY0YZ, 04WY3YZ, 04WY4YZ, 313096, 313097, 313098, 313099.  CPT procedure codes for peripheral surgical revascularization: 35302, 35303, 35304, 35305, 35351, 35355, 35361, 35363, 35371, 35372, 35381, 35480, 35481, 35482, 35483, 35485, 35521, 35537, 35538, 35539, 35540, 35541, 35546, 35548, 35549, 35551, 35556, 35558, 35563, 35565, 35566, 35570, 35571, 35583, 35585, 35587, 35621, 35623, 35641, 35646, 35647, 35651, 35654, 35656, 35661, 35663, 35665, 35666, 35671, 35875, 35876.    ICD9 procedure codes for thrombolysis: 99.10.  ICD10 procedure codes for thrombolysis: 3E03317, 3E04317, 3E05317, 3E06317, 3E08317.  CPT procedure codes for thrombolysis: 37184, 37211, 37213.  ICD9 diagnosis codes for acute myocardial infarction: 410.x0, 410.x1.  ICD10 diagnosis codes for acute myocardial infarction: I21.x, I21.xx.  ICD9 diagnosis codes for ischemic stroke: 433.x1, 434.x1.  ICD10 diagnosis codes for ischemic stroke: I63, I63.x, I63.xx, I63.xxx.  ICD9 diagnosis code for pulmonary embolism: 415.1x.  ICD10 diagnosis codes for pulmonary embolism: T80.0XXA, T81.718A, T81.72XA, T82.817A, T82.818A, I26.90, I26.99.  ICD9 procedure codes for lower extremity amputation above the ankle: 84.13, 84.14, 84.15, 84.16, 84.17.  ICD10 procedure codes for lower extremity amputation above the ankle: 0Y6M0Z0, 0Y6N0Z0, 0Y6H0Z3, 0Y6J0Z3, 0Y670ZZ, 0Y680ZZ, 0Y6C0Z1, 0Y6C0Z3, 0Y6D0Z1, 0Y6D0Z2, 0Y6D0Z3, 0Y6F0ZZ, 0Y6G0ZZ, 0Y6H0Z1, 0Y6H0Z2, 0Y6J0Z1, 0Y6J0Z2, 0Y620ZZ, 0Y630ZZ, 0Y640ZZ.  CPT procedure codes for lower extremity amputation above the ankle: 27590, 27591, 27592, 27598, 27880, 27881, 27882, 27888.  ICD9 diagnosis code for traumatic amputation of a leg: 897.x.  ICD10 diagnosis codes for traumatic amputation of a leg: S78.xxxA, S88.xxxA, where xxx can be any 3-digit number.  ICD9 diagnosis codes for peripheral artery disease: 440.2, 440.20, 440.21, 440.22, 440.23, 440.24, 440.29, 440.3, 440.30, 440.31, 440.32, 440.4, 443.9.  ICD10 diagnosis codes for peripheral artery disease: I70.2, I70.20, I70.201, I70.202, I70.203, I70.208, I70.209, I70.21, I70.211, I70.212, I70.213, I70.218, I70.219, I70.22, I70.221, I70.222, I70.223, I70.228, I70.229, I70.23, I70.231, I70.232, I70.233, I70.234, I70.235, I70.238, I70.239, I70.24, I70.241, I70.242, I70.243, I70.244, I70.245, I70.248, I70.249, I70.25, I70.26, I70.261, I70.262, I70.263, I70.268, I70.269, I70.29, I70.291, I70.292, I70.293, I70.298, I70.299, I70.3, I70.30, I70.301, I70.302, I70.303, I70.308, I70.309, I70.31, I70.311, I70.312, I70.313, I70.318, I70.319, I70.32, I70.321, I70.322, I70.323, I70.328, I70.329, I70.33, I70.331, I70.332, I70.333, I70.334, I70.335, I70.338, I70.339, I70.34, I70.341, I70.342, I70.343, I70.344, I70.345, I70.348, I70.349, I70.35, I70.36, I70.361, I70.362, I70.363, I70.368, I70.369, I70.39, I70.391, I70.392, I70.393, I70.398, I70.399, I70.4, I70.40, I70.401, I70.402, I70.403, I70.408, I70.409, I70.41, I70.411, I70.412, I70.413, I70.418, I70.419, I70.42, I70.421, I70.422, I70.423, I70.428, I70.429, I70.43, I70.431, I70.432, I70.433, I70.434, I70.435, I70.438, I70.439, I70.44, I70.441, I70.442, I70.443, I70.444, I70.445, I70.448, I70.449, I70.45, I70.46, I70.461, I70.462, I70.463, I70.468, I70.469, I70.49, I70.491, I70.492, I70.493, I70.498, I70.499, I70.5, I70.50, I70.501, I70.502, I70.503, I70.508, I70.509, I70.51, I70.511, I70.512, I70.513, I70.518, I70.519, I70.52, I70.521, I70.522, I70.523, I70.528, I70.529, I70.53, I70.531, I70.532, I70.533, I70.534, I70.535, I70.538, I70.539, I70.54, I70.541, I70.542, I70.543, I70.544, I70.545, I70.548, I70.549, I70.55, I70.56, I70.561, I70.56, I70.562, I70.563, I70.568, I70.569, I70.59, I70.591, I70.592, I70.593, I70.598, I70.599, I70.6, I70.60, I70.601, I70.602, I70.603, I70.608, I70.609, I70.61, I70.611, I70.612, I70.613, I70.618, I70.619, I70.62, I70.621, I70.622, I70.623, I70.628, I70.629, I70.63, I70.631, I70.632, I70.633, I70.634, I70.635, I70.638, I70.639, I70.64, I70.641, I70.642, I70.643, I70.644, I70.645, I70.648, I70.649, I70.65, I70.66, I70.661, I70.662, I70.663, I70.668, I70.669, I70.69, I70.691, I70.692, I70.693, I70.698, I70.699, I70.7, I70.70, I70.701, I70.702, I70.703, I70.708, I70.709, I70.71, I70.711, I70.712, I70.713, I70.718, I70.719, I70.72, I70.721, I70.722, I70.723, I70.728, I70.729, I70.73, I70.731, I70.732, I70.733, I70.734, I70.735, I70.738, I70.739, I70.74, I70.741, I70.742, I70.743, I70.744, I70.745, I70.748, I70.749, I70.75, I70.76, I70.761, I70.762, I70.763, I70.768, I70.769, I70.79, I70.791, I70.792, I70.793, I70.798, I70.799, I70.92, I73.9. |
| CVD = cardiovascular disease; MI = myocardial infarction | |

Supplemental Table 5. Incidence rates and hazard ratios for risk for all-cause mortality following hospital discharge for myocardial infarction among patients with Medicare insurance.

|  | **Prior CVD (n=89,920)** | **Diabetes only (n=46,032)** | **CKD only (n=13,459)** | **Diabetes and CKD  (n=13,319)** |
| --- | --- | --- | --- | --- |
| **All-cause mortality*** |  |  |  |  |
| Number of events | 28,900 | 9,716 | 5,425 | 5,171 |
| Incidence Rate (95% CI) | 165 (163, 167) | 118 (116, 120) | 232 (226, 238) | 218 (212, 224) |
| Hazard ratio (95% CI) |  |  |  |  |
| Model 1 | 1 (ref) | 0.89 (0.87-0.92) | 1.10 (1.07-1.14) | 1.35 (1.31-1.39) |
| Model 2 | 1 (ref) | 0.89 (0.87-0.92) | 1.03 (1.00-1.07) | 1.21 (1.18-1.25) |
| CI = confidence interval; CKD = chronic kidney disease; CVD = cardiovascular disease  Incidence rates are presented as per 1,000 person-years. Model 1 includes adjustment for age, sex, race/ethnicity (for patients in the Medicare sample), geographic region of residence and area-level income (for patients in the Medicare sample). Model 2 includes adjustment for Model 1 plus smoking, hypertension, depression, history of heart failure, cardiologist care, endocrinologist care, antihypertensive medication use, statin therapy and intensity, and ezetimibe use. *Medicare cohort only | | | | |

Supplemental Table 6. Incidence rates and hazard ratios of cardiovascular disease events and all-cause mortality following hospital discharge for myocardial infarction stratified by sex.

|  | **Prior CVD** | **Diabetes only** | **CKD only** | **Diabetes and CKD** | **P-interaction** |
| --- | --- | --- | --- | --- | --- |
| **CVD events** |  |  |  |  |  |
| Incidence rate (95% CI) |  |  |  |  |  |
| Women | 133 (131, 136) | 115 (112, 118) | 134 (127, 141) | 175 (167, 183) |  |
| Men | 136 (134, 139) | 105 (102, 108) | 113 (106, 120) | 167 (159, 175) |  |
| Hazard ratio (95% CI) |  |  |  |  |  |
| Women | 1 (ref) | 0.97 (0.93-1.01) | 0.97 (0.92-1.03) | 1.24 (1.17-1.30) | <0.001 |
| Men | 1 (ref) | 0.87 (0.84-0.90) | 0.80 (0.75-0.85) | 1.12 (1.06-1.18) |  |
| **Myocardial infarction** |  |  |  |  |  |
| Incidence rate (95% CI) |  |  |  |  |  |
| Women | 88 (86, 90) | 71 (68, 73) | 95 (89, 100) | 124 (118, 131) |  |
| Men | 80 (78, 82) | 55 (53, 57) | 74 (69, 79) | 109 (103, 115) |  |
| Hazard ratio (95% CI) |  |  |  |  |  |
| Women | 1 (ref) | 0.95 (0.91-0.99) | 1.00 (0.94-1.07) | 1.32 (1.24-1.40) | <0.001 |
| Men | 1 (ref) | 0.80 (0.76-0.84) | 0.82 (0.76-0.89) | 1.16 (1.09-1.24) |  |
| **Coronary heart disease** |  |  |  |  |  |
| Incidence rate (95% CI) |  |  |  |  |  |
| Women | 109 (107, 112) | 96 (93, 99) | 112 (106, 118) | 147 (140, 154) |  |
| Men | 119 (116, 121) | 89 (86, 91) | 96 (90, 102) | 142 (135, 150) |  |
| Hazard ratio (95% CI) |  |  |  |  |  |
| Women | 1 (ref) | 0.98 (0.94-1.02) | 1.00 (0.94-1.06) | 1.27 (1.20-1.34) | <0.001 |
| Men | 1 (ref) | 0.84 (0.81-0.88) | 0.80 (0.75-0.86) | 1.11 (1.04-1.17) |  |
| **Stroke** |  |  |  |  |  |
| Incidence rate (95% CI) |  |  |  |  |  |
| Women | 20 (19, 21) | 16 (15, 17) | 20 (17, 22) | 21 (19, 24) |  |
| Men | 13 (12, 14) | 11 (11, 12) | 14 (11, 16) | 17 (15, 19) |  |
| Hazard ratio (95% CI) |  |  |  |  |  |
| Women | 1 (ref) | 0.95 (0.87-1.04) | 0.87 (0.76-0.99) | 1.04 (0.91-1.19) | 0.438 |
| Men | 1 (ref) | 1.07 (0.96-1.19) | 0.88 (0.74-1.04) | 1.14 (0.97-1.34) |  |
| **Peripheral artery disease** |  |  |  |  |  |
| Incidence rate (95% CI) |  |  |  |  |  |
| Women | 7 (6, 7) | 6 (5, 6) | 5 (4, 6) | 9 (7, 11) |  |
| Men | 7 (6, 7) | 6 (5, 7) | 4 (3, 5) | 11 (9, 13) |  |
| Hazard ratio (95% CI) |  |  |  |  |  |
| Women | 1 (ref) | 0.87 (0.74-1.02) | 0.76 (0.58-0.99) | 1.21 (0.98-1.48) | 0.248 |
| Men | 1 (ref) | 0.98 (0.84-1.13) | 0.57 (0.41-0.78) | 1.37 (1.12-1.68) |  |
| **All-cause mortality*** |  |  |  |  |  |
| Incidence rate (95% CI) |  |  |  |  |  |
| Women | 188 (185, 191) | 134 (131, 138) | 267 (258, 276) | 232 (224, 241) |  |
| Men | 144 (142, 147) | 100 (96, 103) | 190 (182, 199) | 201 (192, 209) |  |
| Hazard ratio (95% CI) |  |  |  |  |  |
| Women | 1 (ref) | 0.93 (0.90-0.96) | 1.09 (1.05-1.14) | 1.25 (1.20-1.30) | <0.001 |
| Men | 1 (ref) | 0.84 (0.81-0.87) | 0.95 (0.91-1.00) | 1.16 (1.11-1.22) |  |
| CI = confidence interval; CKD = chronic kidney disease; CVD = cardiovascular disease Incidence rates are presented per 1,000 person-years. Hazard ratios include adjustment for age, sex, race/ethnicity (for patients in the Medicare sample), geographic region of residence and area-level income (for patients in the Medicare sample), smoking, hypertension, depression, history of heart failure, cardiologist care, endocrinologist care, antihypertensive medication use, statin therapy and intensity, and ezetimibe use. *Medicare cohort only | | | | | |

Supplemental Table 7. Incidence rates and hazard ratios for risk for cardiovascular disease events and all-cause mortality among patients following hospital discharge for myocardial infarction stratified by insulin use.

|  | **Prior CVD (n=89,920)** | **Diabetes only, not taking insulin† (n=34,527)** | **Diabetes only, taking insulin† (n=11,505)** | **CKD only (n=13,459)** | **Diabetes and CKD, not taking insulin‡ (n=7,994)** | **Diabetes and CKD, taking insulin‡ (n=5,325)** |
| --- | --- | --- | --- | --- | --- | --- |
| **CVD events** |  |  |  |  |  |  |
| Number of events | 21,619 | 6,512 | 2,878 | 2,637 | 1,946 | 1,571 |
| Incidence rate (95% CI) | 135 (133, 137) | 99 (97, 101) | 146 (140, 151) | 124 (119, 129) | 152 (146, 159) | 202 (192, 212) |
| Hazard ratio (95% CI) |  |  |  |  |  |  |
| Model 1 | 1 (ref) | 0.78 (0.76-0.80) | 1.14 (1.10-1.19) | 0.86 (0.82-0.89) | 1.06 (1.02-1.11) | 1.42 (1.35-1.49) |
| Model 2 | 1 (ref) | 0.84 (0.82-0.87) | 1.20 (1.15-1.25) | 0.89 (0.85-0.93) | 1.07 (1.02-1.13) | 1.38 (1.31-1.46) |
| **Myocardial infarction** |  |  |  |  |  |  |
| Number of events | 14,350 | 3,925 | 1,800 | 1,894 | 1,408 | 1,154 |
| Incidence rate (95% CI) | 84 (82, 85) | 56 (54, 58) | 84 (80, 88) | 85 (81, 89) | 104 (99, 110) | 138 (130, 146) |
| Hazard ratio (95% CI) |  |  |  |  |  |  |
| Model 1 | 1 (ref) | 0.74 (0.71-0.77) | 1.13 (1.07-1.19) | 0.89 (0.85-0.94) | 1.14 (1.08-1.20) | 1.57 (1.48-1.67) |
| Model 2 | 1 (ref) | 0.80 (0.77-0.83) | 1.17 (1.11-1.24) | 0.92 (0.87-0.96) | 1.13 (1.07-1.19) | 1.48 (1.39-1.58) |
| **Coronary heart disease** |  |  |  |  |  |  |
| Number of events | 18,741 | 5,591 | 2,464 | 2,266 | 1,682 | 1,369 |
| Incidence rate (95% CI) | 114 (113, 116) | 84 (81, 86) | 121 (116, 126) | 105 (100, 109) | 129 (123, 135) | 171 (162, 180) |
| Hazard ratio (95% CI) |  |  |  |  |  |  |
| Model 1 | 1 (ref) | 0.77 (0.75-0.79) | 1.11 (1.06-1.16) | 0.86 (0.82-0.90) | 1.07 (1.02-1.12) | 1.42 (1.34-1.50) |
| Model 2 | 1 (ref) | 0.84 (0.81-0.86) | 1.17 (1.12-1.22) | 0.90 (0.86-0.94) | 1.08 (1.03-1.14) | 1.38 (1.31-1.46) |
| **Stroke** |  |  |  |  |  |  |
| Number of events | 3,019 | 935 | 407 | 401 | 277 | 192 |
| Incidence rate (95% CI) | 16 (16, 17) | 13 (12, 13) | 18 (16, 19) | 17 (15, 18) | 19 (17, 21) | 20 (18, 23) |
| Hazard ratio (95% CI) |  |  |  |  |  |  |
| Model 1 | 1 (ref) | 0.88 (0.82-0.95) | 1.32 (1.18-1.46) | 0.87 (0.79-0.97) | 1.04 (0.92-1.18) | 1.24 (1.07-1.44) |
| Model 2 | 1 (ref) | 0.91 (0.84-0.98) | 1.34 (1.20-1.50) | 0.87 (0.78-0.97) | 1.03 (0.91-1.17) | 1.22 (1.05-1.42) |
| **Peripheral artery disease** |  |  |  |  |  |  |
| Number of events | 1,238 | 364 | 213 | 104 | 116 | 125 |
| Incidence rate (95% CI) | 7 (6, 7) | 5 (4, 5) | 9 (8, 10) | 4 (3, 5) | 8 (6, 9) | 13 (11, 15) |
| Hazard ratio (95% CI) |  |  |  |  |  |  |
| Model 1 | 1 (ref) | 0.72 (0.64-0.81) | 1.29 (1.11-1.50) | 0.65 (0.53-0.79) | 1.07 (0.89-1.30) | 1.70 (1.42-2.05) |
| Model 2 | 1 (ref) | 0.78 (0.69-0.88) | 1.40 (1.19-1.63) | 0.66 (0.54-0.81) | 1.06 (0.87-1.29) | 1.67 (1.38-2.03) |
| **All-cause mortality*** |  |  |  |  |  |  |
| Number of events | 28,900 | 7,008 | 2,708 | 5,425 | 3,072 | 2,099 |
| Incidence rate (95% CI) | 165 (163, 167) | 111 (108, 113) | 142 (137, 147) | 232 (226, 238) | 210 (203, 218) | 230 (220, 240) |
| Hazard ratio (95% CI) |  |  |  |  |  |  |
| Model 1 | 1 (ref) | 0.82 (0.80-0.84) | 1.20 (1.15-1.24) | 1.10 (1.07-1.13) | 1.22 (1.17-1.26) | 1.61 (1.54-1.69) |
| Model 2 | 1 (ref) | 0.82 (0.80-0.85) | 1.17 (1.12-1.21) | 1.03 (1.00-1.06) | 1.11 (1.07-1.15) | 1.43 (1.36-1.50) |
| CI = confidence interval; CKD = chronic kidney disease; CVD = cardiovascular disease  Incidence rates are presented as per 1,000 person-years. Model 1 includes adjustment for age, sex, race/ethnicity (for patients in the Medicare sample), geographic region of residence and area-level income (for patients in the Medicare sample). Model 2 includes adjustment for Model 1 plus smoking, hypertension, depression, history of heart failure, cardiologist care, endocrinologist care, antihypertensive medication use, statin therapy and intensity, and ezetimibe use. *Medicare cohort only  †P-interaction <0.001 for diabetes only by insulin use for each outcome  ‡P-interaction <0.001 for diabetes and CKD by insulin use for CVD events, recurrent MI, coronary heart disease, peripheral artery disease and death  ‡P-interaction = 0.070 for diabetes and CKD by insulin use for stroke | | | | | | |

Supplemental Figure 1. Cumulative incidence of recurrent MI events (top left), coronary heart disease events (top right), stroke events (bottom left), and peripheral artery disease events (bottom right) associated with prior cardiovascular disease, diabetes only, chronic kidney disease only and both diabetes and chronic kidney disease.


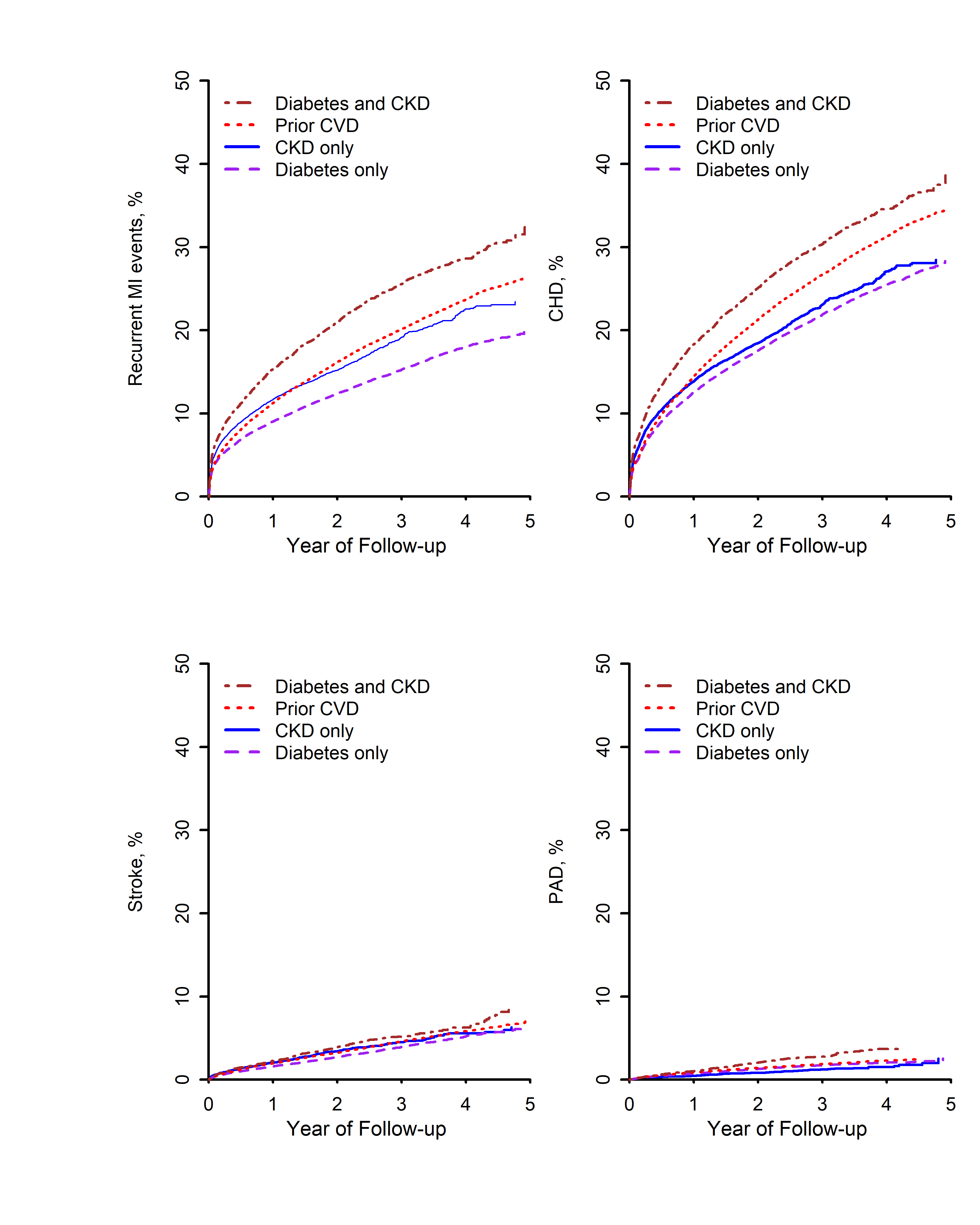


CHD = coronary heart disease; CKD = chronic kidney disease; CVD = cardiovascular disease; MI = myocardial infarction; PAD = peripheral artery disease
